# TASmania: A bacterial Toxin-Antitoxin Systems database

**DOI:** 10.1371/journal.pcbi.1006946

**Published:** 2019-04-25

**Authors:** Hatice Akarsu, Patricia Bordes, Moise Mansour, Donna-Joe Bigot, Pierre Genevaux, Laurent Falquet

**Affiliations:** 1 Department of Biology, University of Fribourg & Swiss Institute of Bioinformatics, Fribourg, Switzerland; 2 Laboratoire de Microbiologie et de Génétique Moléculaires (LMGM), Centre de Biologie Intégrative (CBI), Université de Toulouse, CNRS, UPS, Toulouse, France; University of New South Wales, AUSTRALIA

## Abstract

Bacterial Toxin-Antitoxin systems (TAS) are involved in key biological functions including plasmid maintenance, defense against phages, persistence and virulence. They are found in nearly all phyla and classified into 6 different types based on the mode of inactivation of the toxin, with the type II TAS being the best characterized so far. We have herein developed a new *in silico* discovery pipeline named **TASmania,** which mines the >41K assemblies of the EnsemblBacteria database for known and uncharacterized protein components of type I to IV TAS loci. Our pipeline annotates the proteins based on a list of curated HMMs, which leads to >2^.^10^6^ loci candidates, including orphan toxins and antitoxins, and organises the candidates in pseudo-operon structures in order to identify new TAS candidates based on a guilt-by-association strategy. In addition, we classify the two-component TAS with an unsupervised method on top of the pseudo-operon (pop) gene structures, leading to 1567 “popTA” models offering a more robust classification of the TAs families. These results give valuable clues in understanding the toxin/antitoxin modular structures and the TAS phylum specificities. Preliminary *in vivo* work confirmed six putative new hits in *Mycobacterium tuberculosis* as promising candidates. The TASmania database is available on the following server https://shiny.bioinformatics.unibe.ch/apps/tasmania/.

## Author summary

TASmania offers an extensive annotation of TA loci in a very large database of bacterial genomes, which represents a resource of crucial importance for the microbiology community. TASmania supports i) the discovery of new TA families; ii) the design of a robust experimental strategy by taking into account potential interferences in *trans*; iii) the comparative analysis between TA loci content, phylogeny and/or phenotypes (pathogenicity, persistence, stress resistance, associated host types) by providing a vast repertoire of annotated assemblies. Our database contains TA annotations of a given strain not only mapped to its core genome but also to its plasmids, whenever applicable.

## Introduction

Toxin-antitoxin systems (TAS) were originally known for their involvement in a process known as post-segregational killing (PSK), a plasmid maintenance mechanism based on the differential decay of the products of two plasmid-encoded genes: a toxin gene and its antagonistic antitoxin [1–3]. The current model for TA activation is that under normal growth conditions, the antitoxin efficiently counteracts the toxin negative effects. Yet, under certain stress situations the toxin is released, thus leading to a transient metabolic shutdown and growth arrest. TAS can be acquired from mobile genetic elements such as plasmids or phages, and are also present in core genomes [4]. The ability to be transferred both vertically and horizontally renders any phylogenetic analysis difficult and little is known about the distribution of the TAS among phylum. The work by Wood and his group with artificial toxin derived from endogenous antitoxins (and *vice-et-versa*) highlights the plasticity of ubiquitous TAS and the complexity of their origins [5]. Since the discovery of the PSK, the growing list of TAS related studies has led to a list of more complex (and sometimes controversial) roles for TAS. To name a few, TAS are involved in cell suicide following a phage abortive infection [6] or nutritional stress [7], in regulating biofilm dynamics [8] and in bacterial persistence [9–11]. Some studies even show that chromosomal TAS can counteract PSK [12].

All TAS toxins are proteins that target a variety of essential biological processes (*e*.*g*., membrane integrity, translation, replication) and they are divided in groups based on the nature and mechanism of action of the cognate antitoxin [13]. Currently there are six types of TAS described in the literature. In the type I family, an ncRNA antitoxin (generally in antisense of the toxin gene) inhibits the translation of the toxin mRNA. Typical examples of type I TAS are the hok/sok systems [3]. Type II TAS, which constitute the most commonly studied family, are composed of an antitoxin protein that binds directly to the toxin protein and inhibits its activity. Some toxins target DNA replication [14], or affect the cell membrane integrity by phosphorylating peptidoglycan precursors [15], while others have acetyltransferase activity [16,17], or are kinases that target the translation elongation factor EF-Tu [18,19]. Yet, many type II toxins are ribonucleases that i) cleave target mRNAs in a ribosome-dependent manner [20] or ii) cleave free mRNA [21], and they can also target non coding RNA [22,23]. Type III is a more recent addition, with ToxN/ToxI as a reference member [6] and more families added later by the pioneering work from Salmond’s group [24]. The type III toxin is a nuclease that cleaves a broad range of mRNA and RNA, while the antitoxin is a small non-coding RNA that binds directly to the toxin protein, thus inhibiting its action. In type IV there is no direct interaction between the toxin and antitoxin components. Here the antitoxin counteracts the toxin by competing with its targets, like cytoskeleton proteins [25]. Type V currently has so far only a single member, the GhoT/GhoS system [26], in which the antitoxin itself is an endoribonuclease protein that targets the toxin mRNA [27]. Type VI TAS are grouped TA systems that involve a third partner. This partner promotes the toxin decay in *trans* [28] or the antitoxin stability in *cis* [29].

The ubiquity of the TAS and the diversity of their functions open question about their potential interactions in *trans*. Numerous publications suggest that it may be between noncognates from same families [12,30–32] or between noncognates from different TAS types [33,34]. On the other hand, other data suggest isolated TA units [35]. The Laub group used co-evolution study of protein-protein interactions to show that paralogous ParD/ParE pairs are highly specific in their operon cognates [36]. Nevertheless, their model of promiscuous intermediates still leaves room for interactions in *trans*. Finally, most of the TAS studies focus on the canonical TAS that are usually found in a configuration with the antitoxin gene being upstream of the toxin gene, with few TAS families presenting a reversed order [4,37]. Alternative structures have been mentioned by van Melderen and her group, which highlights the existence of orphan TA loci [38]. So far, TAS screening approaches usually skip the multigene TA systems, despite known tripartite TAS [29,39–41] and TAS modules inserted within operons [7,42].

Validated and predicted TAS are collected in the TADB2 database [43]. TADB2 focuses mainly on type II TAS that were mined from the literature (N = 105 TA loci) and from previous published screens (N = 6088 TA loci) extracted from 870 bacteria and archaea genomes. The 6088 TA loci were predicted using Blastp on 126 genomes [37] or PSI-Blast searches with validated literature datasets [44]. A few of them were additionally combined with known operon structure obtained from STRING [45]. TADB2 also includes a search tool called TAFinder (http://202.120.12.133/TAfinder/index.php) combining homologous search and operon structure module filters [43]. TAFinder uses Blastp searches with the TADB2 dataset and HMM searches with 108 Toxin HMMs and 201 Antitoxin HMMs to select the TA loci. These loci are then filtered using protein size (by default >30aa and <300aa) and intergenic distance (by default from -20nt to +150nt). TADB2 and TAFinder are very stringent in their criteria to minimize false positives.

Our primary goal is to provide the microbiology community with a largely extended database of the type I to type IV (and potentially type V to VI as side hits) toxin and antitoxin loci. We also propose an objective annotation of the TA independently of the cognate components. With the current nomenclature based on the identification of the toxin cognate, the antitoxin would “inherit” the toxin family name. This can be misleading and ignores the modularity of TA cognates. Instead, our method allows the discovery of unexpected combinations of toxin and antitoxin families. We include a “guilt-by-association” approach in our pipeline, similarly to methods developed by others [38,44]. The large dataset of genomes enables us to apply phylogenetic comparisons.

## Results

### EnsemblBacteria assemblies

The EnsemblBacteria database (Rel. 33 Nov. 2016) contains N = 41'610 genomic assemblies that correspond to N = 23'921 unique taxonomic identifiers (taxonomy ids), indicating a high degree of redundancy in the assemblies. At least one hit was found for N = 40'993 assemblies present at least one hit with the TASmania HMM scan, of which N = 22'950 correspond to unique taxonomy ids. A closer look at the taxonomy ids shows that 40% of the genomic assemblies belong to the *Proteobacteria* phylum and 34% to the *Firmicutes* phylum, these two groups making up three quarters of the database (S1 Fig). The *Actinobacteria* and *Bacteroidetes* phyla represent 12% and 3% of the assemblies, respectively. The remaining 11% of the assemblies correspond to N = 72 other phyla and/or unclassified bacteria.

### TASmania strategy

**Fig 1:**
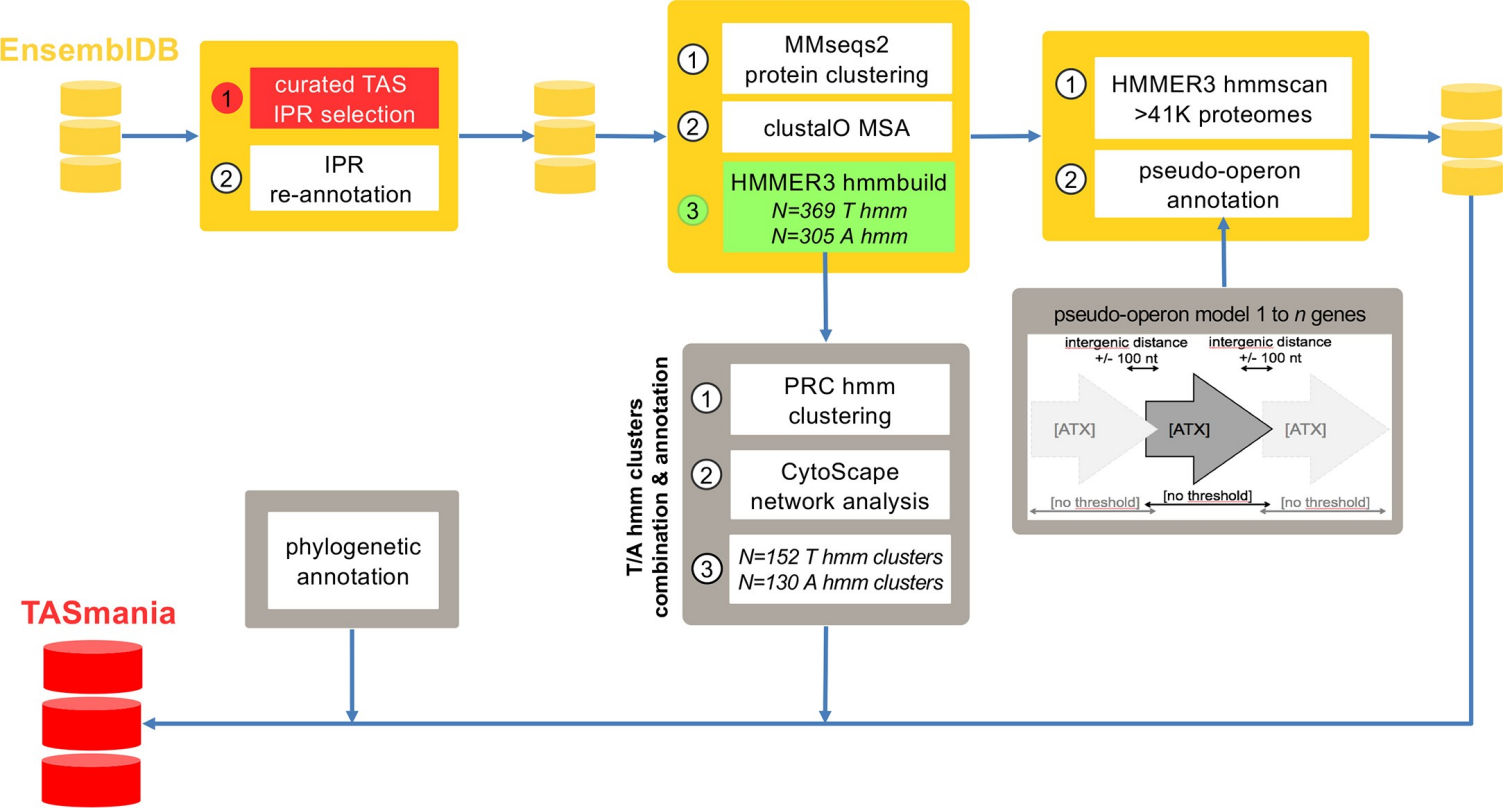
Overview of the pipeline to build the TASmania database. The different steps include: downloading EnsemblBacteria, updating the InterPro annotation, selecting the proteins matching an arbitrary list of reference TAS IPR, building the corresponding HMM profiles and scanning the proteomes. In parallel, we structure target genomes into pseudo-operons and include phylum information. Finally, we add extra value to TASmania by clustering the HMM profiles into larger families for TA combinations analysis.

TASmania is based on the pipeline summarized in Fig 1. Briefly, the strategy relies on TA HMM profiles built from an initial set of proteins annotated with TA InterPro (IPR) (S1 Table). This critical initial set is a known limitation affecting other methods like TADB2 or TAFinder and might lead to missing families. From the protein clustering we obtain N = 369 toxin HMM profiles (with at least 10 unique protein sequences) and N = 305 antitoxin HMM profiles (with at least 10 unique protein sequences). From the theoretical N = 369*305 = 112’545 possible combinations in canonical AT/TA operons, we only observe N = 2’600 HMM profile combinations.

We combine the HMM profiles into larger HMM clusters by similarity. This allows to decrease the number of toxin HMM profiles (N = 369) and antitoxin HMM profiles (N = 305) combinations to plot. When using clustered HMM profiles (N = 152 clusters for toxin HMM profiles and N = 130 clusters for antitoxin HMM profiles), we go from theoretical N = 152*130 = 19’760 combinations to only N = 1’567 observed pairs. Thus, grouping the HMM profiles into clusters allows a decrease of ∼40% in the number of combinations and reduces potential redundancy of certain HMM profiles. We always keep the link between HMM profiles and their clusters. We call each of these clusters TASMANIA.T1 to TASMANIA.T152 (T1 to T152) for the toxins, and TASMANIA.A1 to TASMANIA.A130 (A1 to A130) for the antitoxins. We enhance the value of the putative TA hits by structuring the loci into pseudo-operons and including phylogenetics information. A given combination of two clusters within pseudo-operon is dubbed “popTA”. Finally, for reverse-compatibility with the current TA nomenclature, we also include a nearest Pfam annotation for a given HMM profile and cluster (S2 Table). More details are given in Materials and Methods section.

### TASmania hits global statistics

**Fig 2:**
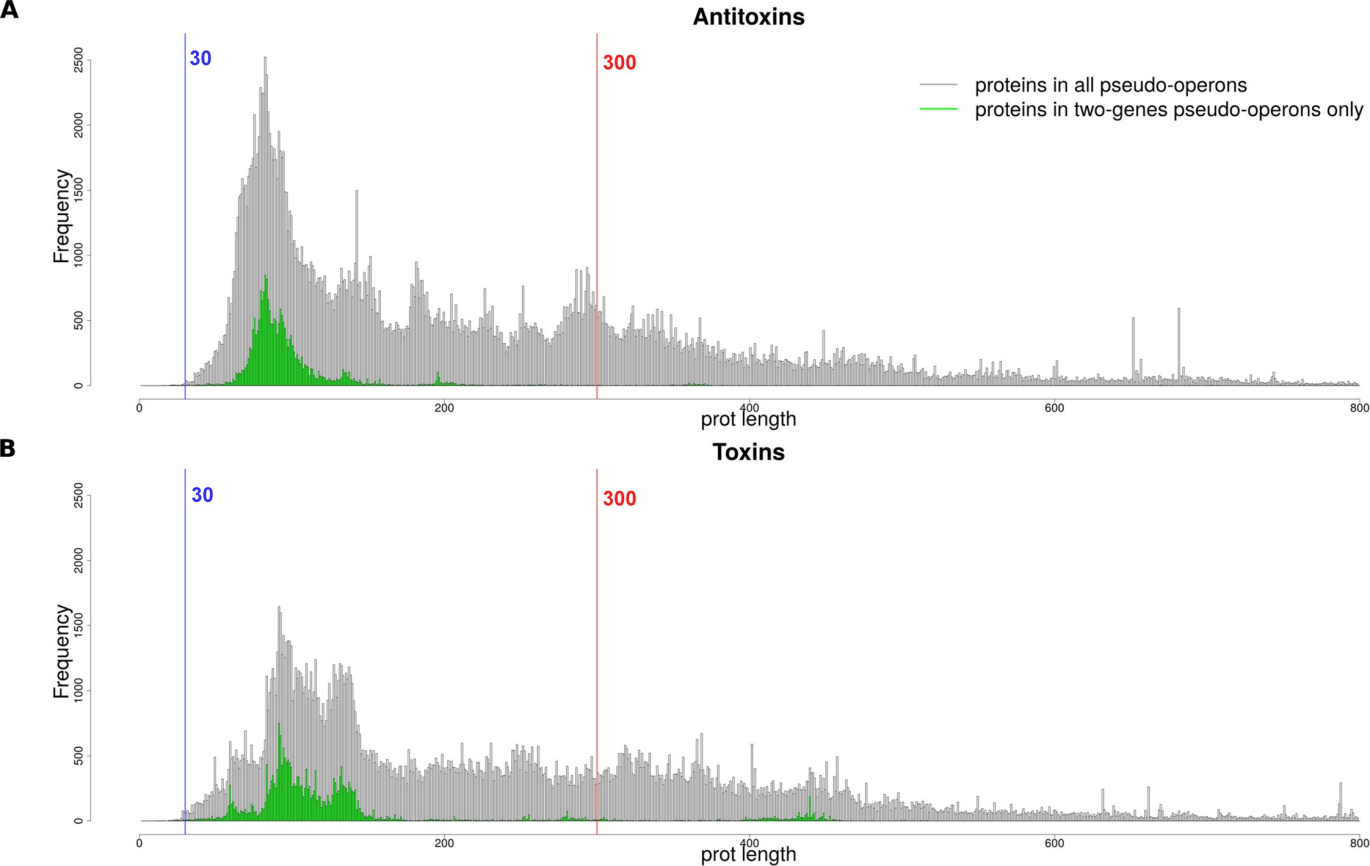
Unique proteins length distribution of TASmania putative hits. (A) Antitoxins length distribution (in amino acids). (B) Toxins length distribution (in amino acids). Blue and red vertical lines correspond to default thresholds used by TAfinder.

After scanning EnsemblBacteria with the HMM profiles, we obtain N = 1'155'070 putative toxin gene hits, corresponding to N = 228'074 unique toxin protein sequences; and N = 1'283'761 putative antitoxin genes hits, corresponding to N = 270'733 unique antitoxin protein sequences. In total, the putative toxin or antitoxin hits correspond to N = 2'298'903 unique pseudo-operons containing TA modules (including redundant ones). A phylogenetic analysis of the TA hits distribution shows that *Cyanobacteria* are very TA-rich and are the most common phylum in the top 200 most TA-enriched genomes (S2 Fig). Our method does not use a protein length filtering, thus allowing for discovery. The protein length distribution of the putative toxin and antitoxin hits confirms previous results [46], as shown in Fig 2. We can see that the absence of length thresholding allows the discovery of more putative TAs (right tail of the distributions). When focusing on the canonical—*i*.*e*., the two-gene T->A or A->T modules—the protein length distribution mimicks the previously published data by narrowing the proteins length into the 30–210 residues window used by [46]. This effect is most probably due to the bias of annotation favouring AT/TA modules. However, as can be seen in green on Fig 2, some toxin and antitoxins of the canonical AT/TA modules exceed the 210 aa limit from [46] and 300 aa from [43].

**Fig 3:**
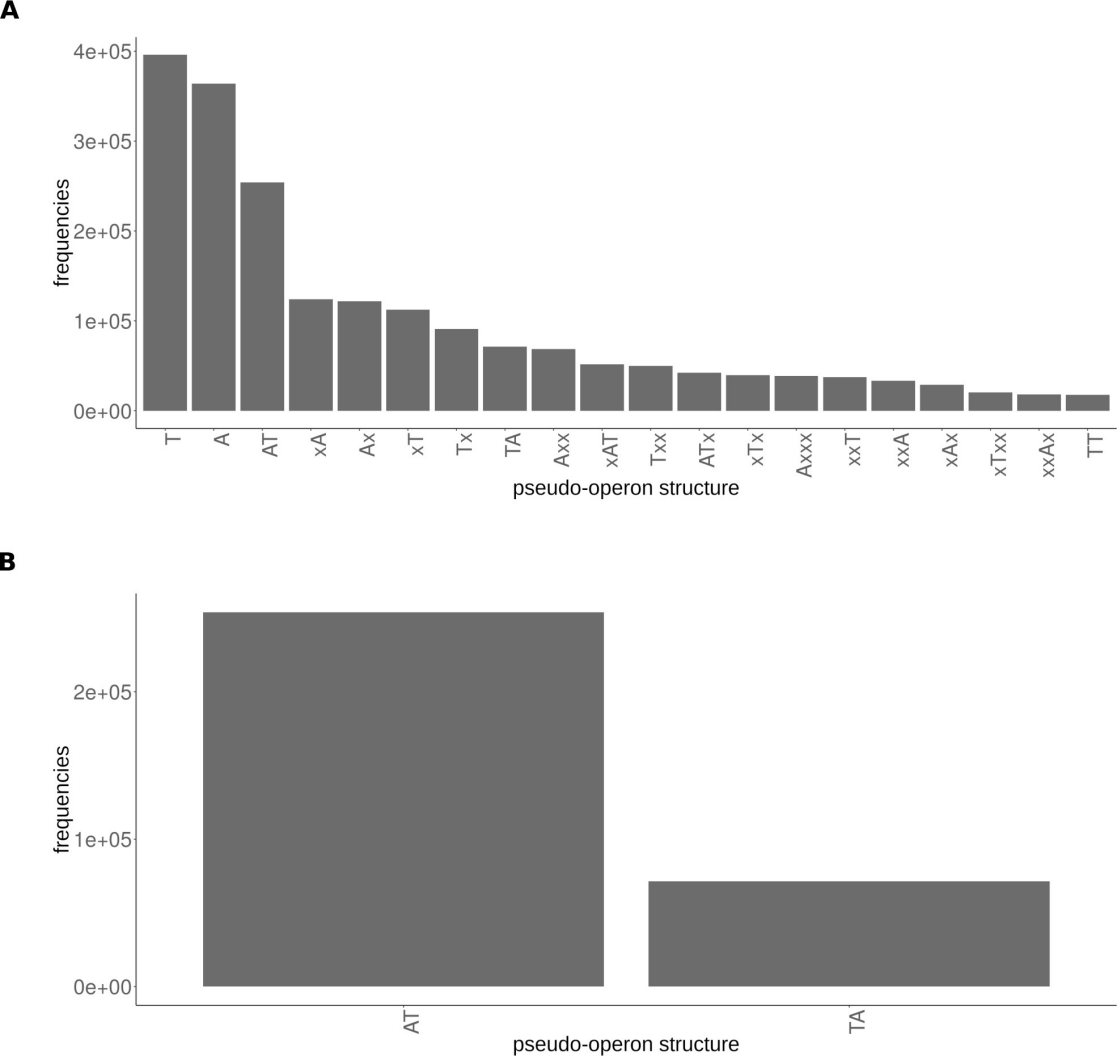
Pseudo-operon types distribution. (A) All hits from the TASmania (only the 20 most frequent pseudo-operons structures are shown). (B). Canonical hits only (two-genes T->A or A->T modules) highlighting the higher abundance of the A->T module type versus the T->A type.

The distribution of the pseudo-operon structures of the HMM scan hits in Fig 3A i) indicates that TAS can be multi-cistronic organisation, not uniquely bi-cistronic.; ii) confirms that the A->T module type is more common than the T->A type and iii) shows the existence of many “orphan” hits, *i*.*e*., a toxin or antitoxin gene as single-gene pseudo-operon. These hits could be either true orphaned T’s or A’s, and/or false positives and/or could be due to the mis-annotation of the operons and/or potentially type I or type III toxins as we cannot detect the ncRNA with our current method. The prevalence of the A->T type is highlighted when comparing only canonical two-genes structures (Fig 3B).

### TASmania performance

**Fig 4:**
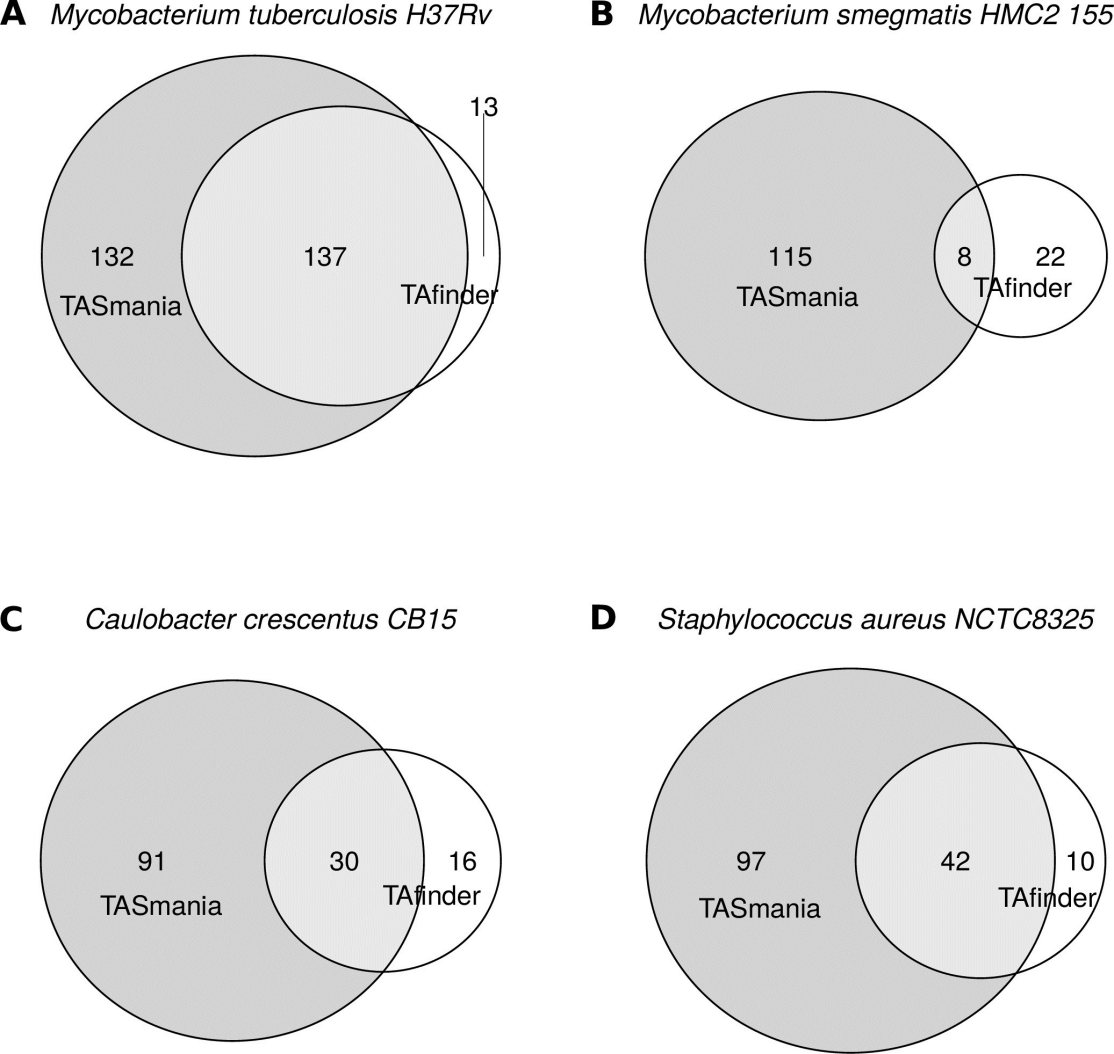
Comparison of TASmania and TAfinder hits. Using *M*.*tuberculosis* as a proof-of-principle, a list of manually curated, new and promising TASmania-specific hits is shown in Table 1, compared to the results obtained by TAfinder on the same genomes. (A) Mycobacterium tuberculosis H37Rv. (B) Mycobacterium smegmatis HMC2 155. (C) Caulobacter crescentus CB15. (D) Staphylococcus aureus NCTC8325. These TASmania-specific TA hits correspond mostly to: i) type I or type IV systems; ii) orphan loci; iii) guilt-by-association “x” loci iv) unusual combinations (“TT”, “AA”). This confirms that our strategy of not filtering out any unusual TAS operon structures or protein lengths allows us to be more discovery-orientated. Including the guilt-by-association “x” cognates is also useful when looking for uncharacterized TAS families.

We compared TASmania putative TAS hits with the ones proposed by TAfinder. Since we cannot download the entire datasets from this webtool, we used a few reference model strains as a proof of principle: *Mycobacterium tuberculosis H37Rv* (*M*.*tuberculosis*), *Mycobacterium smegmatis MC*^*2*^*155* (*M*.*smegmatis*), *Caulobacter crescentus CB15* (*C*.*crescentus*) and *Staphylococcus aureus NCTC8325* (*S*.*aureus*). The putative hits were manually downloaded from these websites and compared against TASmania hits (Fig 4). These data show that TASmania covers most of TAfinder hits and gives many other putative candidates (Fig 4 and S3 Table).

**Table 1:** TASmania hits missed by TAfinder. Some putative *M*.*tuberculosis* TAS are shown. For a complete automated list of hits missed by TAfinder, see S3 Table. Experimentally validated toxins are flagged with a “ab” superscripts. The qualifier “interpro_only” describes proteins that are not found by our HMMs, but had an InterPro hit of the primary IPR list.

ensembl gene id	gene description	protein length	nearest Pfam identifier	nearest Pfam description	TAS info	operon id	operon structure
*Rv0078A*^*a*^^*b*^	Hypothetical protein	197	interpro_only	interpro_only	T	43	TT
*Rv0078B*	Conserved protein	68	SymE_toxin	Toxin SymE, type I toxin-antitoxin system	T	43	TT
*Rv0207c*^*a*^^*b*^	Conserved hypothetical protein	242	interpro_only	interpro_only	T	119	xTx
*Rv0208c*	Hypothetical methlytransferase (methylase)	263	guilt_by_association	guilt_by_association	x	119	xTx
*Rv0229c*^*a*^	Possible conserved membrane protein with PIN domain	226	PIN	PIN domain	T	132	xT
*Rv0230c*	Hypothetical protein	326	guilt_by_association	guilt_by_association	x	132	xT
*Rv0268c*	Hypothetical protein	169	PhdYeFM_antitox	Antitoxin Phd_YefM, type II toxin-antitoxin system	A	150	xA
*Rv0269c*^*a*^^*b*^	Conserved hypothetical protein	397	guilt_by_association	guilt_by_association	x	150	xA
*Rv0277c*	Possible toxin VapC25. Contains PIN domain.	142	PIN	PIN domain	T	157	T
*Rv0366c*^*a*^^*b*^	Conserved hypothetical protein	197	Zeta_toxin	Zeta toxin	T	207	xAATx
*Rv0367c*	Hypothetical protein	129	ParD_like	ParD-like antitoxin of type II bacterial toxin-antitoxin system	A	207	xAATx
*Rv0456A*	Possible toxin MazF1	93	PemK_toxin	PemK-like, MazF-like toxin of type II toxin-antitoxin system	T	248	xxT
*Rv0456B*	Possible antitoxin MazE1	57	guilt_by_association	guilt_by_association	x	248	xxT
*Rv0569*^*a*^	Conserved protein	88	interpro_only	interpro_only	T	304	Tx
*Rv0570*	Probable ribonucleoside-diphosphate reductase (large subunit) NrdZ (ribonucleotide reductase	692	guilt_by_association	guilt_by_association	x	304	Tx
*Rv0634A*	Unknown protein	83	VapB_antitoxin	Bacterial antitoxin of type II TA system, VapB	A	339	A
*Rv1044*	Conserved hypothetical protein	207	AbiEi_4	Transcriptional regulator, AbiEi antitoxin	A	551	AT
*Rv1045*	Hypothetical protein	293	AbiEii	Nucleotidyl transferase AbiEii toxin, Type IV TA system	T	551	AT
*Rv2016*^*a*^^*b*^	Hypothetical protein	191	HicA_toxin	HicA toxin of bacterial toxin-antitoxin	T	1059	TA
*Rv2017*	Transcriptional regulatory protein	346	HTH_3	Helix-turn-helix	A	1059	TA
*Rv2165c*^*a*^	Conserved protein	396	guilt_by_association	guilt_by_association	x	1128	Axxx
*Rv2166c*	Conserved protein	143	MraZ	MraZ protein, putative antitoxin-like	A	1128	Axxx
*Rv2405*	Conserved protein	189	PemK_toxin	PemK-like, MazF-like toxin of type II toxin-antitoxin system	T	1271	T
*Rv2514c*^*a*^^*b*^	Conserved hypothetical protein	153	interpro_only	interpro_only	T	1324	xT
*Rv2515c*	Conserved hypothetical protein	415	guilt_by_association	guilt_by_association	x	1324	xT
*Rv3662c*^*a*^	Conserved hypothetical protein	256	interpro_only	interpro_only	T	1914	xxxT
*Rv3663c*	Probable dipeptide-transport ATP-binding protein ABC transporter DppD	548	guilt_by_association	guilt_by_association	x	1914	xxxT

^a^toxin genes that have been tested experimentally

^b^toxin genes that have been tested experimentally and validated as toxic and rescued by the antitoxin cognate

Looking closely at the TAfinder hits missed by TASmania, the module *Rv2653c/Rv2654c* in *M*.*tuberculosis* H37Rv seems to encode prophage proteins, with no IPR annotation, hence their absence from TASmania (S4 Table). This module could be a real TAS and if this hypothesis happens to be confirmed experimentally, they will be added to TASmania profiles. The remaining TAfinder hits missed by TASmania fall into the transcriptional regulators (*e*.*g*., ArsR, LysR, TetR, MarR), transposases and uncharacterized proteins categories. It is difficult to evaluate if these loci are true TA missed by TASmania or false positives from TAfinder.

### Experimental confirmation of putative TASmania hits

**Fig 5:**
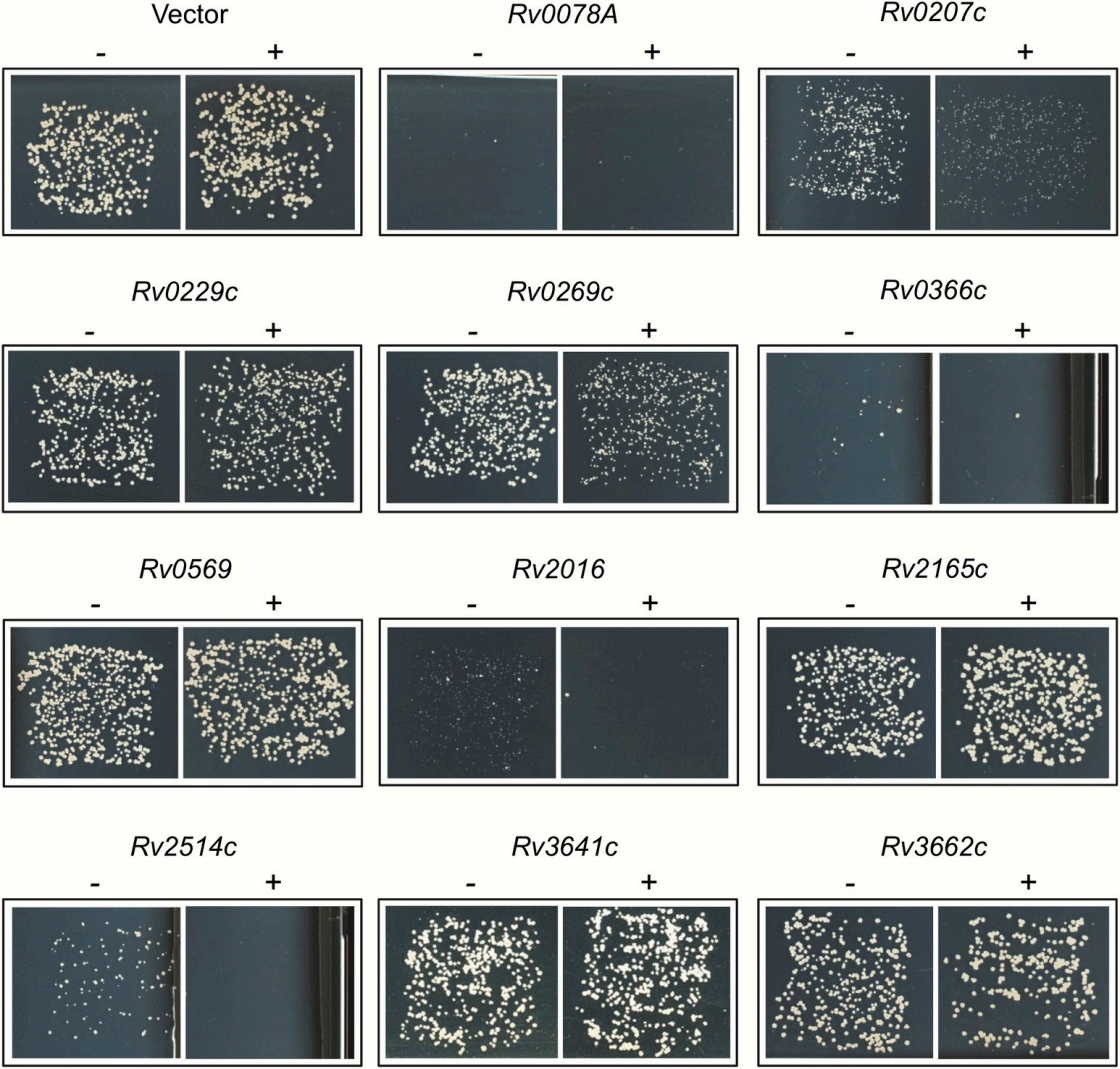
Expression of putative toxins in *M*.*smegmatis*. *M*.*smegmatis* strain MC^2^155 was freshly transformed with pLAM12-based constructs expressing the putative toxin encoding genes of *M*.*tuberculosis* identified in this work, namely *Rv0078A*, *Rv0207c*, *Rv0229c*, *Rv0269c*, *Rv0366c*, *Rv0569*, *Rv2016*, *Rv2165c*, *Rv2514c*, *Rv3641c* and *Rv3662c*. Transformants were plated on LB agar supplemented with kanamycin, without (-) or with 0.2% acetamide inducer (+). Plates were incubated 3 days at 37°C.

Although it is technically not possible to assess the overall rate of false positives in the TASmania-specific hits, the *in vivo* analysis performed on some TASmania candidates shows promising results. We investigated whether some of the putative TA systems of *M*.*tuberculosis* identified by TASmania were indeed *bona fide* new TA systems. We selected 11 putative TA systems that are not found by TAfinder or TADB2 and asked whether expression of their putative toxins could affect growth of the closely related *M*.*smegmatis* strain MC^2^155. Putative toxin encoding genes were cloned into the pLAM12 vector under the control of an acetamide inducible promoter, transformed into MC^2^155 and incubated for 3 days at 37°C on kanamycin agar plates without or with 0.2% acetamide inducer. Under these conditions we found that six out of eleven putative toxins affected *M*.*smegmatis* growth, with four of them exhibiting a robust toxicity, namely *Rv0078A*, *Rv0366c*, *Rv2016* and *Rv2514c*, and two only inducing a slow growth phenotype, namely *Rv0207c* and *Rv0269c* (Fig 5). These results suggest that these six genes could encode toxins of new or uncharacterized TA systems in *M*.*tuberculosis*, thus further extending the long list of TA in this bacterium [47].

**Fig 6:**
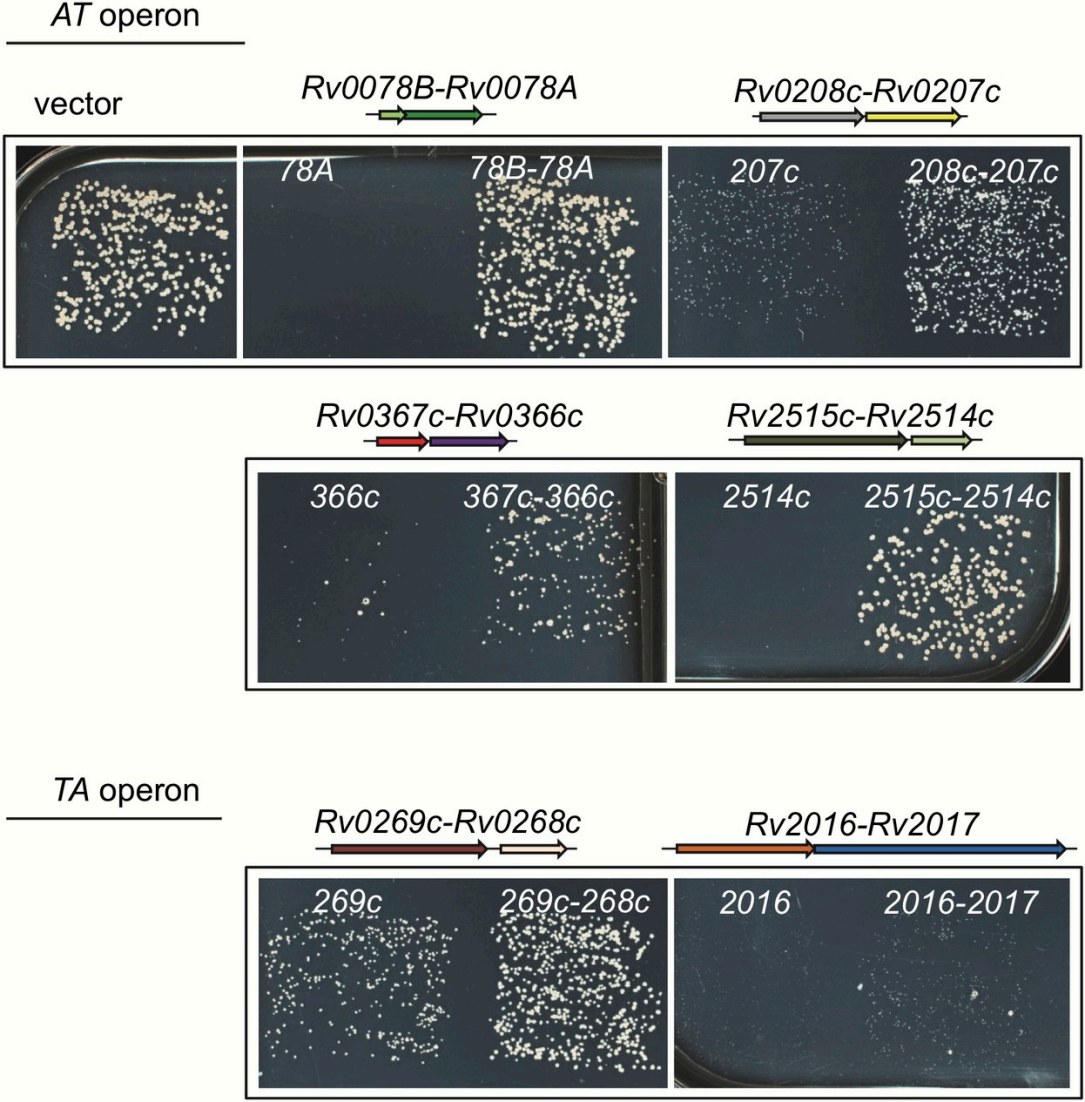
Six putative TA of *M*.*tuberculosis* validated by rescue test in *M*.*smegmatis*. *M*.*smegmatis* strain MC^2^155 was freshly transformed with pLAM12-based constructs expressing the putative toxic genes of *M*.*tuberculosis* (*Rv0078A*, *Rv0207c*, *Rv0269c*, *Rv0366c*, *Rv2016* and *Rv2514c*) either alone or as an operon together with their respective putative antitoxin genes, namely *Rv0078B/Rv0078A*, *Rv0208c/Rv0207c*, *Rv0269c/Rv0268c*, *Rv0367c/Rv0366c*, *Rv2016/Rv2017* and *Rv2515c/Rv2514c*. Transformants were plated on LB agar supplemented with kanamycin and acetamide inducer (0.2%), except for *Rv0366c and Rv0367c/Rv0366c*, which shows suppression by the putative antitoxin only in the absence of acetamide. Plates were incubated for three days at 37°C.

In order to investigate whether these toxic genes are part of *bona fide* TA systems, the six corresponding TA operons composed of the putative toxin encoding genes and of the putative cognate antitoxin genes were cloned in pLAM12 vector, transformed in MC^2^155 and their effect on bacterial growth was monitored as in Fig 6. Note that 4 out of these 6 putative TA systems are in antitoxin first, toxin second (AT orientation), and the last two in toxin first, antitoxin second gene organization (TA orientation) (Fig 6).

We found that in all cases bacterial growth could be rescued by the presence of the putative antitoxin genes in all cases, although to various levels (Fig 6). *Rv0078B/Rv0078A* (A->T) and *Rv2515c/Rv2514c* (A->T) operons both support the *in silico* prediction of putative TAS: the high toxicity of the putative toxin expressed alone is inhibited by the co-expression of the putative cognate antitoxin. *Rv0078B/Rv0078A* (A->T) is a very interesting case. Remarkably, although *Rv0078B* acts as an antitoxin and rescues the toxicity of *Rv0078A*, TASmania HMM scan flags *Rv0078B* as a putative toxin from the cluster T52 (nearest Pfam SymE_toxin type I). *Rv0078A* is also flagged as a toxin via its IPR annotation (IPR014942 AbiEii toxin type IV). This unexpected predicted “TT” pair could be the signature of a new family of TAS, with *Rv0078B* being a potential example of a TAS cognate that “switched” function [4]. T52 hits like *Rv0078B* are found in diverse pseudo-operons structures, although T52 should in theory be a toxin of type I and therefore rather appears in pseudo-operons looking like orphans (“T”). *M*.*tuberculosis* presents only a single pseudo-operon with T52 hit, while it is absent from *M*.*smegmatis* and appears in N = 34 different loci in *Thalassomonas actiniarum*. In the latter, T52 hits are all orphan toxins, suggesting that, in this species at least, T52 looks more like a classical SymE-like toxin type I (the antitoxin cognate being a ncRNA, it cannot be annotated currently by TASmania).

On the other hand, *Rv0208c/Rv0207c* and *Rv0269c/Rv0268c* are both putative TAS operons with the toxin exhibiting a weak toxicity when expressed in *M*.*smegmatis*. This could be due to various reasons, including missing/divergent *M*.*tuberculosis* toxin targets in *M*.*smegmatis*, potential cross-interactions in *trans* with the cognate antitoxins of other similar TAS, a poorly expressed toxin in *M*.*smegmatis*, a non-essential toxin target or a target not required under the growth conditions tested. *Rv0269c/Rv0268c* is a TAS in T->A conformation, with the antitoxin *Rv0268c* annotated as a A24 (nearest Pfam family PhdYeFM_antitox), while *Rv0269c* is proposed as a guilt-by-association toxin. In *M*.*tuberculosis*, only *Rv0268c* is found as a A24 hit, but many other loci (N = 12) belong to PhdYeFM_antitox clusters (A24, A9, A27, A81, A94, A100). *Rv0269c/Rv0268c* is interesting since it is in a T->A configuration, which is unusual for the PhdYeFM antitoxin. Homologies suggest that *Rv0269c* is related to proteins with a DNA polymerase/primase/ligase domain. Therefore *Rv0269c/Rv0268c* is a puzzling pair worth deeper investigation. Whether these two systems are *bona fide* TA pairs remains to be investigated.

*Rv0367c/Rv0366c* (A->T) is a putative TA couple where both loci are hit by TASmania HMM profiles belonging to the A123 (nearest Pfam ParD_like) and T70 (nearest Pfam Zeta_toxin) clusters, respectively. The combination A123.T70 (nearest Pfam ParD_like.Zeta_toxin) could represent a new TAS family, since the canonical zeta toxin is described in the literature as the cognate of epsilon antitoxin. In the TASmania database, most of T70 clusters hits appear as paired with A49 and A123 clusters (both with nearest Pfam ParD_like).

Finally, in the case of *Rv2016 (T144 nearest Pfam HicA_toxin)*, which is highly toxic when expressed in *M*.*smegmatis*, we could also detect an effective but very limited suppression of toxicity in the presence of the putative antitoxin gene *Rv2017* (A32 nearest Pfam HTH_3). Whether this is due to the genetic organization with the toxin and/or to the lack of a chaperone partner is unknown [48]. All together, these experimental validations of TASmania *in silico* predictions show how our database can be a very powerful tool in discovering unexpected TAS families.

### T/A HMM profile clusters combinations (popTAs)

For clarity and reproducibility, we focus on the two-genes modules to study the toxin and antitoxin clusters co-occurrence within the pseudo-operons, *i*.*e*., popTAs. In order to minimize bias introduced by the overrepresentation of certain phylogenetic groups over others (see S1 Fig), we apply a correction to cluster counts with the weight of each phylum in the database. Out of the theoretical N = 152*130 = 19'760 possible combinations, we find N = 1'522 popTAs, independently of their T->A or A->T orientation; and N = 1'567 popTAs if the orientation is taken into account.

#### Modularity of the clusters

**Fig 7:**
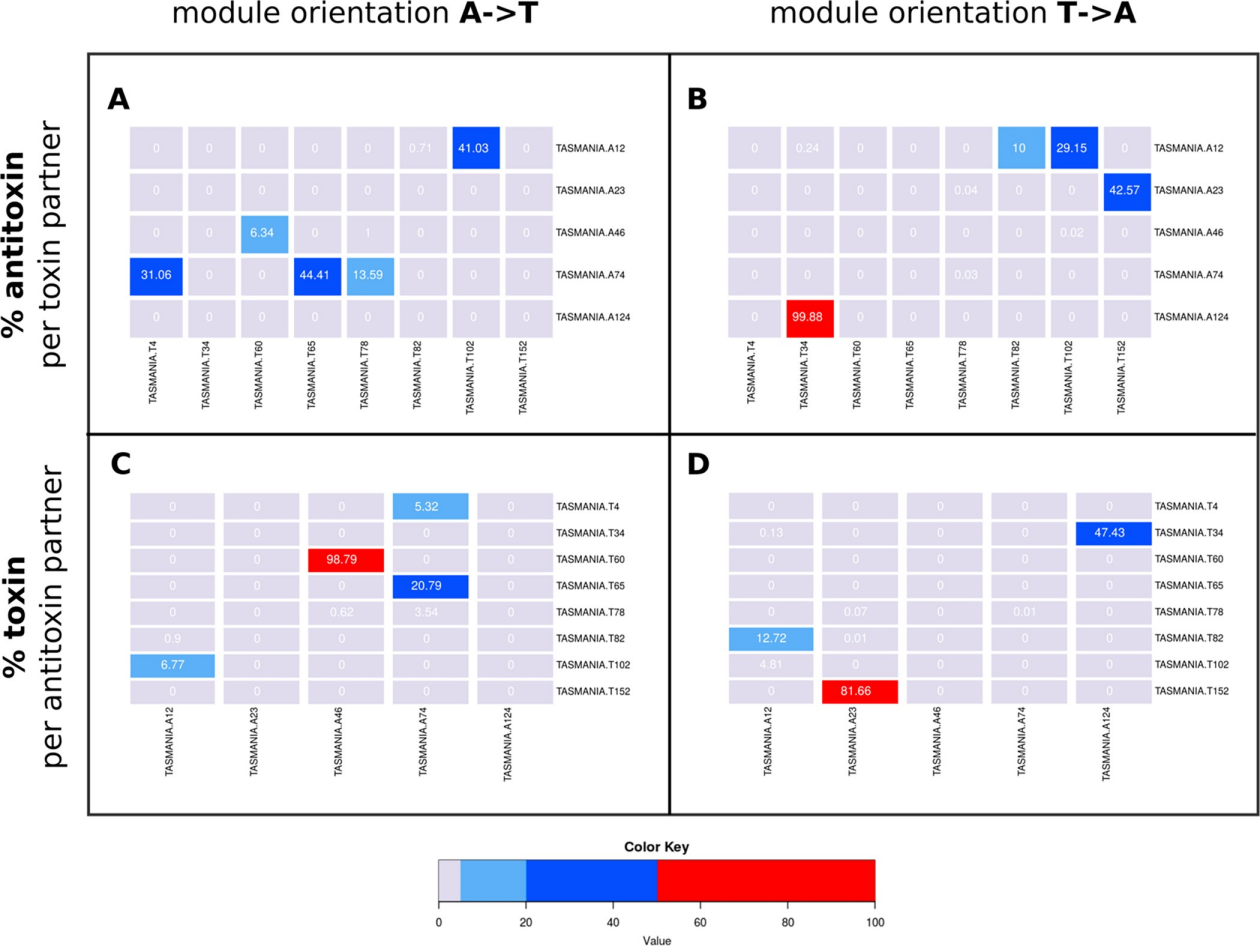
Examples of co-occurrence of toxin and antitoxin clusters within two-genes pseudo-operons (popTAs). The color key correspond to percentages (%), given in each cell. (A) Antitoxin clusters in A->T orientation, and their relation to toxin clusters. For instance, the modular A74 antitoxin cluster has three main cognates the T4, T65 and T78 toxin clusters: A74.T4 (31.06% of A74 popTAs) (nearest Pfam PhdYeFM_antitox.YafQ_toxin), A74.T65 (44.41% of A74 popTAs) (nearest Pfam PhdYeFM_antitox.PIN) and A74.T78 (13.59% of A74 popTAs) (nearest Pfam PhdYeFM_antitox.ParE_toxin). (B) Antitoxin clusters in T->A orientation, and their relation to toxin clusters. For instance, the bi-directional A12 antitoxin cluster’s main toxin cognate is T102, as in T102.A12 (29.15% of A12 popTAs) (nearest Pfam HicB_lk_antitox.HicA_toxin). A restrictive antitoxin cluster is also highlighted with A124 co-occurring mainly with T34 as in T34.A124 (99.88% of A124 popTAs) (nearest Pfam BrnT_toxin.BrnA_antitoxin). (C) Toxin clusters in A->T orientation, and their relation to antitoxin clusters. The restrictive T60 toxin cluster and its association with A46 in A46.T60 (98.79% of T60 popTAs) (nearest Pfam CcdA.CcdB) is given as example. (D) Toxin clusters in T->A orientation, and their relation to antitoxin clusters. T152 is also a quite restrictive toxin cluster that mostly has A23 as the main antitoxin cognate, as in T152.A23 (nearest Pfam HigB-like_toxin.HTH_3). The complete co-occurrence is shown in S3 Fig and described in S5 Table.

The modularity was already partially described by [38,44] and the directionality by others among which [4,37,49]. We refine these concepts by adding the “asymmetry” property: an antitoxin cognate’s favourite toxin is not necessarily reciprocal. An interesting discovery is the imbalance in the degree of modularity and directionality preference of some HMM clusters summarized in co-occurrence heatmaps (Fig 7).

These data confirm the A->T, versus the T->A direction bias. Also they highlight that i) given the actual data, certain clusters seem to be rather unidirectional, such as T60 toxin cluster (nearest Pfam CcdB) observed only in A->T pairs; ii) while others can be found in either configuration A->T or T->A, for example A12.T102 (nearest Pfam HicB_lk_antitox.HicA_toxin, Fig 7A) corresponds to 41% of the popTA containing the A12 cluster, and T102.A12 (nearest Pfam HicA_toxin.HicB_lk_antitox, Fig 7B) counts for 29% of the A12 popTA; iii) certain clusters are restrictive in the range of their pairing cognate (*e*.*g*., T60, T13, see Fig 7C and S5 Table), while others are more “opportunistic” and can associate with a broader range of cognates (*e*.*g*., T74, see Fig 7C and S5 Table). Thus, toxin and antitoxin clusters have diverse degrees of modularity. Typically, the A74 cluster (Pfam PhdYeFM_antitox) pairs in *cis* with several distinct toxin clusters: ~44% of A74 containing popTA are combined with T65 (Pfam PIN) as in A74.T65, ~31% with T4 (Pfam YafQ_toxin) as in A74.T4, ~14% with T78 (Pfam ParE_toxin) as in A74.T78 (Fig 7A and S5 Table). Antitoxins from the A74 cluster could therefore regulate not only their *cis* toxin genes, but also other toxins in *trans*, if multiple TAS are present in a given bacterial genome (S6 Table). This degree of modularity of T and A clusters can be used to identify early on any putative TAS network in a given genomic background. *In silico* data from TASmania with different genomes from distinct phyla confirm these potential interferences (S6 Table). Similarly, the A12 cluster is not only bi-directional as in A12.T102 and T102.A12 (Fig 7A), but is also modular and is present in various configurations within a same genomic background, in many different genomes and phyla (S6 Table).

While some clusters are highly modular, others have only been observed with a restricted cognate family so far. For instance, toxin cluster T60 is observed mainly with A46 (99% of the popTA where T60 is present), as in the A46.T60 popTA (nearest Pfam CcdA.CcdB, Fig 7C). Similarly, the A124 cluster—whose nearest Pfam profile is a BrnA_antitoxin—presents a low modularity value: almost all of popTA with a A124 cluster contain T34 cluster (Pfam BrnT_toxin), as in the T34.A124 (Fig 7B). In the T->A oriented popTAs, the T152 toxin cluster is another example of a less modular cluster, since it has for main cognate the A23 antitoxin cluster (82% of the popTA where T152 is present, S5 Table), as in T152.A23 popTA (nearest Pfam HigB-like_toxin.HTH_3, Fig 7D and S5 Table).

#### Phylum-specific popTAs versus “universal” popTAs

**Fig 8:**
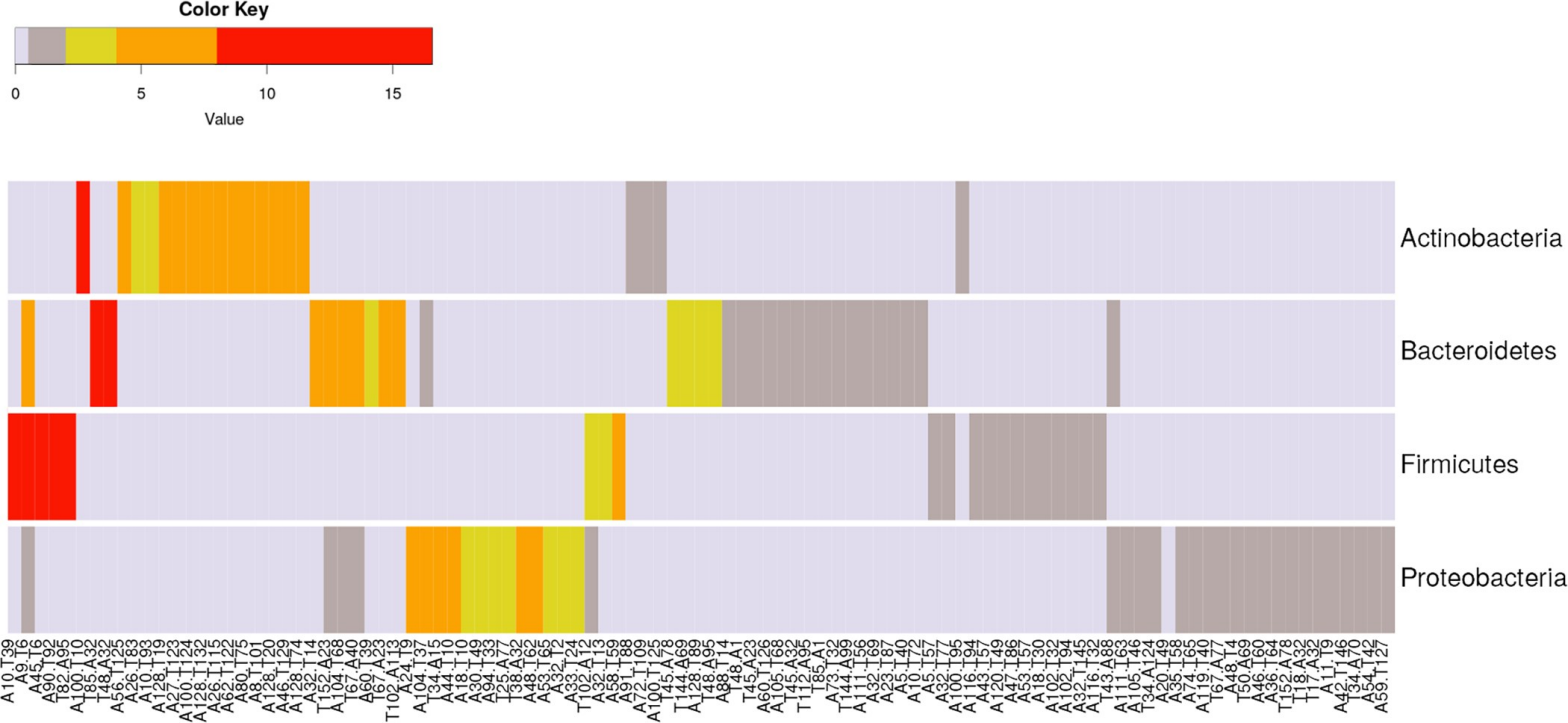
popTAs across phyla. The most abundant popTAs in relative numbers are specific to each phylum.

A previous study looking at the TAS distribution among taxa revealed non-overlapping patterns between *Actinobacteria*, *Firmicutes* and *Proteobacteria* (heatmap of Fig 3, [13]). To estimate the popTAs distribution among phyla in TASmania, we computed the relative abundance of each popTA in a given phylum. We did not observe popTAs with a minimum relative abundance of 1% across the four main phyla simultaneously. Only the popTA A9.T6 (nearest Pfam PhdYeFM.YoeB) is found in 3 phyla simultaneously at a relative abundance greater than 1% (Fig 8).

In the case of *Actinobacteria*, the most abundant popTAs are A100.T10, A56.T127, A26.T85, A10.T95, A128.T19, A27.T125, A100.T126, A128.T134, A26.T117, A62.T124, A80.T77, A8.T103, A128.T20, A46.T131 and A128.T76. The toxin cognate clusters of these popTAs belong mainly to the Pfam PIN family (N = 12/15), while the antitoxin component cluster is more diverse. These popTA are compiled in Table 2.

**Table 2:** popTA across phyla. List of popTA and their corresponding nearest Pfam annotation of the most enriched popTA in each phylum.

popTA	Nearest pfam	phylum
A100.T10	PhdYeFM_antitox.PIN	*Actinobacteria*
A56.T125	PhdYeFM_antitox.PIN	*Actinobacteria*
A26.T83	VapB_antitoxin.PIN	*Actinobacteria*
A10.T93	AbiEi_4.AbiEii	*Actinobacteria*
A128.T19	RHH_1.PIN	*Actinobacteria*
A27.T123	PhdYeFM_antitox.ParE_toxin	*Actinobacteria*
A100.T124	PhdYeFM_antitox.PIN	*Actinobacteria*
A128.T132	RHH_1.PIN	*Actinobacteria*
A26.T115	VapB_antitoxin.PIN	*Actinobacteria*
A62.T122	RHH_1.PemK_toxin	*Actinobacteria*
A80.T75	RHH_1.PIN	*Actinobacteria*
A8.T101	MazE_antitoxin.PIN	*Actinobacteria*
A128.T20	RHH_1.PIN	*Actinobacteria*
A46.T129	CcdA.PIN	*Actinobacteria*
A128.T74	RHH_1.PIN	*Actinobacteria*
T85.A32	Gp49.HTH_3	*Bacteroidetes*
T48.A32	Gp49.HTH_3	*Bacteroidetes*
A32.T14	HTH_3.HipA_C	*Bacteroidetes*
T152.A23	HigB-like_toxin.HTH_3	*Bacteroidetes*
A104.T68	ParD_antitoxin.ParE_toxin	*Bacteroidetes*
T67.A40	HigB-like_toxin.HTH_3	*Bacteroidetes*
A60.T39	AbiEi_4.AbiEii	*Bacteroidetes*
T67.A23	HigB-like_toxin.HTH_3	*Bacteroidetes*
T102.A113	HicA_toxin.HicB_lk_antitox	*Bacteroidetes*
T45.A78	HigB_toxin.HTH_3	*Bacteroidetes*
T144.A69	HicA_toxin.HicB	*Bacteroidetes*
A128.T89	RHH_1.PIN	*Bacteroidetes*
T48.A95	Gp49.HicB_lk_antitox	*Bacteroidetes*
A10.T39	AbiEi_4.AbiEii	*Firmicutes*
A45.T6	PhdYeFM_antitox.YoeB_toxin	*Firmicutes*
A90.T92	PhdYeFM_antitox.YoeB_toxin	*Firmicutes*
T82.A95	HicA_toxin.HicB_lk_antitox	*Firmicutes*
T102.A12	HicA_toxin.HicB_lk_antitox	*Firmicutes*
A32.T13	HTH_3.Zeta_toxin	*Firmicutes*
A58.T59	RelB.ParE_toxin	*Firmicutes*
A24.T9	PhdYeFM_antitox.ParE_toxin	*Proteobacteria*
A104.T37	ParD_antitoxin.ParE_toxin	*Proteobacteria*
T34.T15	BrnT_toxin.BrnA_antitoxin	*Proteobacteria*
A44.T10	MazE_antitoxin.PIN	*Proteobacteria*
A18.T10	MazE_antitoxin.PIN	*Proteobacteria*
A30.T49	MazE_antitoxin.PemK_toxin	*Proteobacteria*
A94.T33	PhdYeFM_antitox.Fic	*Proteobacteria*
T25.A77	HigB_toxin.HTH_3	*Proteobacteria*
T38.A32	RelE.HTH_3	*Proteobacteria*
A48.T62	RelB.YafQ_toxin	*Proteobacteria*
A53.T65	RHH_3.PIN	*Proteobacteria*
A32.T2	HTH_3.HipA_C	*Proteobacteria*
A33.T24	PrlF_antitoxin.Toxin_YhaV	*Proteobacteria*

The most common popTAs found in *Bacteroidetes* belong rather to the T->A configuration (N = 9/13), whose main representatives in the literature are HigB.HigA and HicA.HicB [4,37]. The phylum distribution of the popTAs further confirms the complex picture of the TAS cognate combinations and their modularity, as introduced in Fig 7. Some popTAs like A26.T83 and A26.T115 (both a Pfam VapB_antitoxin.PIN from *Actinobacteria*) are an expected TA combination (VapB.VapC). But other popTAs highlight the modularity of certain families that can be “mixed-and-matched” in *cis*. For example, popTA A8.T101 (*Actinobacteria*), A44.T10 (*Proteobacteria*) and A18.T10 (*Proteobacteria*)—all corresponding to the nearest Pfam profiles pair MazE_antitoxin.PIN—clearly show that MazE-like families of antitoxins can have VapC-like toxin cognates instead of the expected MazF ones (Tables 2 and S6). This confirms previously published data of genomic arrangements in *M*.*tuberculosis* showing MazE antitoxins paired with VapC toxins [31]. The popTA A46.T129 in *Actinobacteria* (Pfam CcdA.PIN) is also another interesting result highlighting an unexpected TA cognates combination. The conventional view of a toxin family (*e*.*g*., PIN or VapC) pairing always with a specific antitoxin family (*e*.*g*., VapB) does not hold any longer.

#### Sequence comparisons of popTA

**Fig 9:**
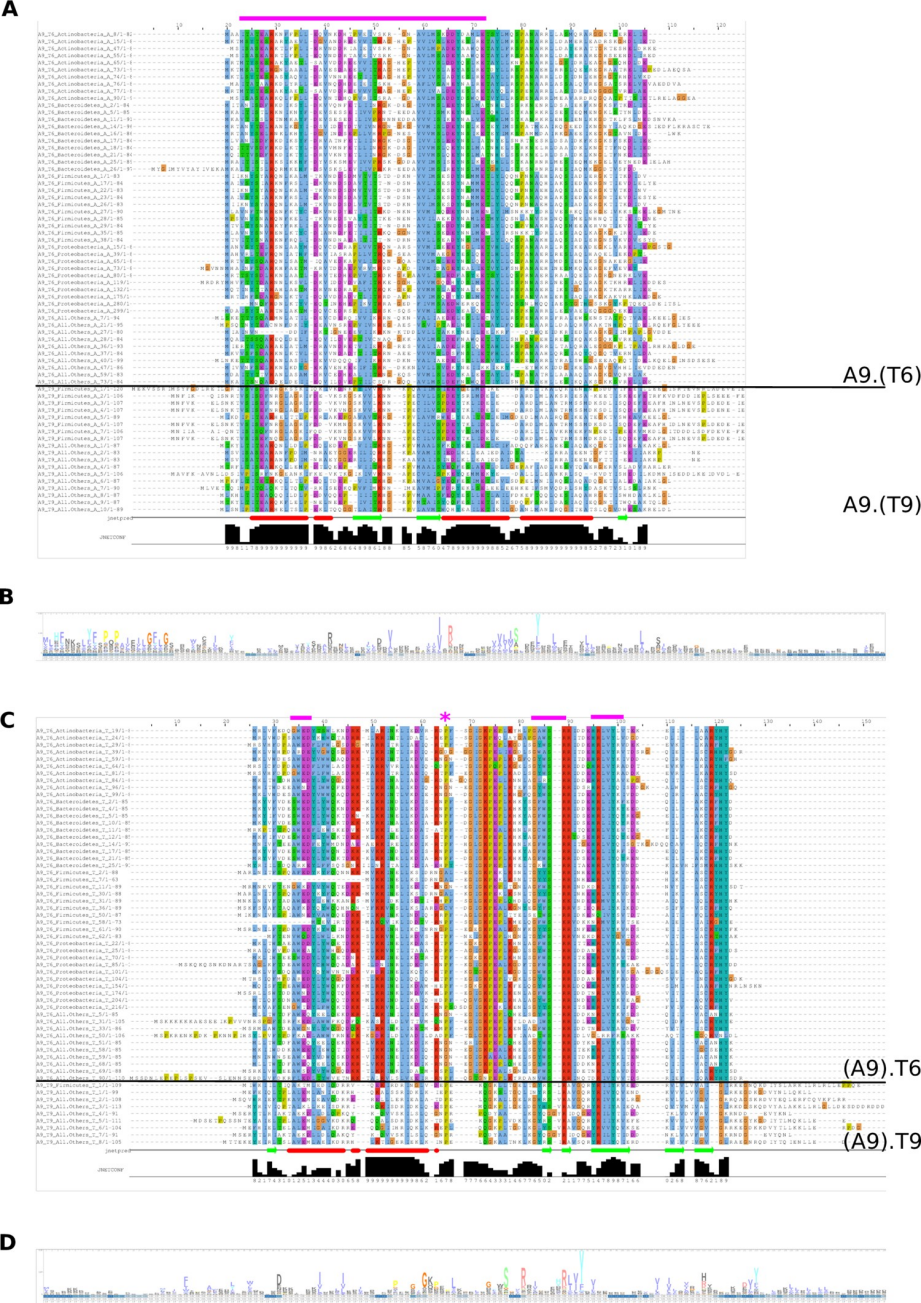
A9 cluster as example of cluster modularity. (A) Multiple sequence alignment of A9 antitoxin cluster (nearest Pfam PhdYeFM_antitox) proteins that are associated with T6 (nearest Pfam YoeB_toxin) or T9 (nearest Pfam ParE_toxin) toxin clusters. (B) HMM profile from antitoxin A9 cluster proteins, in A9.T6 and A9.T9 popTA. (C) Multiple sequence alignment of T6 or T9 toxin clusters proteins associated with A9 antitoxin cluster. (D) HMM profile from toxin T6 and T9 clusters proteins, in A9.T6 and A9.T9 popTA. Note: for clarity, only a subset of sequences are drawn. The magenta bars and stars highlight the conserved residues and regions.

We use A9.T6—a popTA that is found across several phyla (Fig 8), to illustrate the sequence basis behind cluster modularity (Fig 9). Antitoxin proteins from the A9 cluster (nearest Pfam PhdYeFM_antitox) associated with T6 (nearest Pfam YoeB_toxin) and T9 (nearest Pfam ParE_toxin) toxin clusters are relatively conserved, which is expected since all these proteins were hit by the HMM profiles from the A9 cluster (Fig 9A). It is remarkable though that antitoxin proteins from very diverse phyla share such a high degree of conservation. However, the higher variability though in the C-terminus domain of these A9 antitoxins proteins suggests that this region could be involved in the T6 versus T9 binding specificities. Regarding the toxin cognates in T6 and T9 clusters, they are clearly different enough to belong to distinct clusters, but they do share some conserved key residues that could play a role in the interaction with the A9 antitoxin (magenta bars and stars in Fig 9C).

### Granularity of TAS annotations

The popTA features highlight the potential issues that the TA annotations can produce. In the current way toxins and antitoxins are annotated, namely by giving priority to the toxin for naming the antitoxin, many inconsistencies are created. For example in *M*.*tuberculosis*, several antitoxins are annotated as a “VapB” while the TASmania HMM profiles hitting these antitoxins belong to diverse Pfam families like PhdYeFM, ribbon-helix-helix (RHH), CopG or MazE (Table 3).Therefore, we here propose a more objective and systematic annotation of the toxins and antitoxins based on cluster identifiers, rather than misleading functional names inferred from cis-occurrence.

**Table 3:** Granularity of the traditional TAS annotations. Example of *M*.*tuberculosis* H37Rv with some so-called VapB.VapC TA pairs. We propose a more objective nomenclature of the TAS based on the HMM profiles clusters. Note that all VapCs shown here have a PIN Pfam annotation, however their TASMANIA.T*n* (T*n*) is split into multiple sub-clusters emphasizing the diversity of the PIN domains. In contrast, their associated so-called VapB-like antitoxins have very diverse Pfam annotations, but consistent TASMANIA.A*n* (A*n*) clusters.

ensembl gene id	gene description	hmm cluster Pfam annotation	popTA
*Rv2009*	Antitoxin VapB15	VapB_antitoxin	A26.T115
*Rv2010*	Toxin VapC15	PIN	A26.T115
*Rv2526*	Possible antitoxin VapB17	VapB_antitoxin	A26.T83
*Rv2527*	Possible toxin VapC17	PIN	A26.T83
*Rv0596c*	Possible antitoxin VapB4	PhdYeFM_antitox	A100.T10
*Rv0595c*	Possible toxin VapC4	PIN	A100.T10
*Rv0626*	Possible antitoxin VapB5	PhdYeFM_antitox	A100.T10
*Rv0627*	Possible toxin VapC5	PIN	A100.T10
*Rv3181c*	Conserved protein	PhdYeFM_antitox	A100.T125
*Rv3180c*	Hypothetical alanine rich protein	PIN	A100.T125
*Rv3385c*	Possible antitoxin VapB46	PhdYeFM_antitox	A100.T124
*Rv3384c*	Possible toxin VapC46. Contains PIN domain.	PIN	A100.T124
*Rv0581*	Possible antitoxin VapB26	RHH_1	A128.T132
*Rv0582*	Possible toxin VapC26. Contains PIN domain.	PIN	A128.T132
*Rv2104c*	Possible antitoxin VapB37	RHH_1	A128.T19
*Rv2103c*	Possible toxin VapC37. Contains PIN domain.	PIN	A128.T19
*Rv2601A*	Possible antitoxin VapB41	RHH_1	A128.T74
*Rv2602*	Possible toxin VapC41. Contains PIN domain.	PIN	A128.T74
*Rv3321c*	Possible antitoxin VapB44	RHH_1	A128.T20
*Rv3320c*	Possible toxin VapC44. Contains PIN domain.	PIN	A128.T20
*Rv0616A*	Possible antitoxin VapB29	RHH_1	A80.T75
*Rv0617*	Possible toxin VapC29. Contains PIN domain.	PIN	A80.T75
*Rv0550c*	Possible antitoxin VapB3	CcdA	A46.T129
*Rv0549c*	Possible toxin VapC3	PIN	A46.T129
*Rv0599c*	Possible antitoxin VapB27	MazE_antitoxin	A8.T101
*Rv0598c*	Possible toxin VapC27. Contains PIN domain.	PIN	A8.T101
*Rv2595*	Possible antitoxin VapB40	SpoVT_C	A91.T88
*Rv2596*	Possible toxin VapC40. Contains PIN domain.	PIN	A91.T88
*Rv0660c*	Possible antitoxin MazE2	RHH_1	A62.T122
*Rv0659c*	Toxin MazF2	PemK_toxin	A62.T122
*Rv2865*	Antitoxin RelF	PhdYeFM_antitox	A27.T123
*Rv2866*	Toxin RelG	ParE_toxin	A27.T123

### Discovery of TAS candidate protein families

The guilt-by-association approach [38,44] allows the discovery of previously undescribed protein families. This strategy relies on the non-targeted cognate loci of TASmania hits in two-genes operons—“xT”, “Tx”, “Ax” and “xA”. For convenience we focus on xT/Tx starting by collecting and pooling the protein sequences corresponding to the “x” cognates of toxins HMM hits in TASmania. These x cognates are loci that do not have any previous IPR annotation corresponding to known TAS families, nor are they picked up by any of HMM profiles. But they have a toxin as direct neighbour gene, identified by TASmania HMM profile(s) and/or direct IPR annotation. As a proof of principle, we screen all the “x” genes having as neighbour a toxin T cognate, in two-genes pseudo-operons “xT” and “Tx” (we dub these two types of pairs as “popTx”, independently of the orientation). We obtain N = 24’377 unique protein sequences that could potentially belong to new uncharacterized antitoxins. We build and cluster the HMM profiles using the same procedure as for TASmania (see Methods below). These putative new antitoxin families are summarized in Table 4.

**Table 4:** Putative new antitoxin families inferred from the “guilt-by-association” loci. The guilt-by-association loci next to toxin (as in “xT”, “Tx”) hits are collected and analysed for putative new antitoxin families.

putative new antitoxin group	nearest Pfam equivalent	popTx	popTx Pfam annotation	TAS orientation	strains (examples)	ensembl gene id pairs (examples)	taxa where found
A*371	VraX	A*371.T143	VraX.PemK_toxin	AT	staphylococcus_aureus_subsp_aureus_vrs1 (Firmicutes)	*MQA_02482/MQA_02483*	*Firmicutes*
A*77	Glyoxalase	T32.A*77	YafQ_toxin.Glyoxalase	TA	actinomyces_sp_s6_spd3 (Actinobacteria)	*HMPREF1627_07880/HMPREF1627_07885*	*Actinobacteria*
A*190	Colicin_Pyocin	A*190.T4	Colicin_Pyocin.YafQ_toxin	AT	helicobacter_pylori_51 (Proteobacteria)	*KHP_0917/KHP_0916*	*Proteobacteria*
A*237	Antirestrict	A*237.T3	Antirestrict.CbtA_toxin	AT	yersinia_pekkanenii (Proteobacteria)	*ERS008529_03983/ERS008529_03984*	*Proteobacteria*
A*2	Response_reg	T5.A*2	Cpta_toxin.Response_reg	TA	microbacterium_sp_leaf351 (Actinobacteria)	*ASG00_11075/ASG00_11080*	*Actinobacteria*, *Firmicutes*, *Proteobacteria*, *Others*
					enterococcus_gilvus_atcc_baa_350_gca_000407545 (Firmicutes)	*I592_01041/I592_01042*	
					bradyrhizobium_sp_btai1 (Proteobacteria)	*Bbta_6074/Bbta_6075*	
					ardenticatena_maritima_gca_001306175 (Others)	*SE16_06610/SE16_06605*	
A*72	AP_endonuc_2	A*72.T47	AP_endonuc_2.ParE_toxin	AT	xanthomonas_euvesicatoria (Proteobacteria)	*XEU66b_20875/XEU66b_20880*	*Proteobacteria*
		A*72.T56			pseudorhodoferax_sp_leaf267 (Proteobacteria)	*ASF43_11980/ASF43_11975*	
		A*72.T63			pseudomonas_amygdali_pv_aesculi (Proteobacteria)	*ALO90_03560/ALO90_03559*	
		A*72.T91			sinorhizobium_meliloti_2011 (Proteobacteria)	*SM2011_a6409/SM2011_a1770*	
		A*72.T136			acetobacter_tropicalis (Proteobacteria)	*AtDm6_0779/AtDm6_0780*	
A*8	HTH_3	T25.A*8	HigB_toxin.HTH_3	TA	klebsiella_pneumoniae_subsp_pneumoniae_kpnih27 (Proteobacteria)	*KPNIH27_29720/KPNIH27_29715*	*Bacteroidetes*, *Proteobacteria*, *Others*
		T45.A*8			pedobacter_glucosidilyticus (Bacteroidetes)	*PBAC_02340/PBAC_02350*	
A*1	HTH_3	A*1.T2	HTH_3.HipA_C	AT	clavibacter_michiganensis_subsp_sepedonicus (Actinobacteria)	*CMS2889/CMS2890*	*Actinobacteria*, *Bacteroidetes*, *Firmicutes*, *Proteobacteria*, *Others*
		A*1.T14			porphyromonas_cangingivalis (Bacteroidetes)	*HQ34_07435/HQ34_07440*	
					roseburia_faecis (Firmicutes)	*M72_00971/M72_00981*	
					pseudomonas_batumici (Proteobacteria)	*UCMB321_1156/UCMB321_1157*	
		A*1.T16			corynebacterium_sp_nml_130206 (Actinobacter)	*Clow_01920/Clow_01921*	
A*1	HTH_3	T14.A*1	HipA_C.HTH_3	TA	sulfurospirillum_multivorans_dsm_12446 (Proteobacteria)	*SMUL_0082/SMUL_0081*	*Proteobacteria*
		T17.A*1			acinetobacter_baumannii_naval_57 (Proteobacteria)	*ACINNAV57*.*A0098/ACINNAV57*.*A0099*	
A*1, A*8	HTH_3	T21.A*1	ParE_toxin.HTH_3	TA	haemophilus_influenzae (Proteobacteria)	*CK45_05580/CK45_05585*	*Proteobacteria*
		T78.A*1			agrobacterium_arsenijevicii (Proteobacteria)	*RP75_28995/RP75_29000*	
		T123.A*1			ralstonia_solanacearum_fqy_4 (Proteobacteria)	*F504_3323/F504_3322*	
		T9.A*8			pseudomonas_brassicacearum_subsp_brassicacearum_nfm421 (Proteobacteria)	*PSEBR_c2g85/PSEBR_a3991*	
A*1	HTH_3	T18.A*1	RelE.HTH_3	TA	streptomyces_purpurogeneiscleroticus (Actinobacteria)	*ADL19_02735/ADL19_02740*	*Actinobacteria*, *Firmicutes*, *Proteobacteria*, *Others*
					anaerovibrio_lipolyticus (Firmicutes)	*NZ47_10830/NZ47_10835*	
					citrobacter_freundii (Proteobacteria)	*MC47_06900/MC47_06895*	
		T38.A*1			mycobacterium_tuberculosis_gca_001376955 (Actinobacteria)	*ERS053691_04167/ERS053691_04168*	
					escherichia_vulneris_nbrc_102420 (Proteobacteria)	*EV102420_16_00320/EV102420_16_00330*	
A*27	RHH_1	A*27.T61	RHH_1.ParE_toxin	AT	propionibacterium_acnes_hl043pa2 (Actinobacteria)	*HMPREF9571_00118/HMPREF9571_00119*	*Actinobacteria*, *Firmicutes*, *Proteobacteria*, *Others*
					listeria_booriae (Firmicutes)	*EP57_10595/EP57_10600*	
					salmonella_enterica_subsp_enterica_gca_001431385 (Proteobacteria)	*AO411_29300/AO411_29305*	
A*6	Phage_integrase	T58.A*6	CcdB.Phage_integrase	TA	escherichia_coli_10_0833 (Proteobacteria)	*EC100833_5859/EC100833_5860*	*Proteobacteria*
A*6	Phage_integrase	A*6.T57	Phage_integrase.PemK_toxin	AT	geobacillus_thermoglucosidasius_c56_ys93 (Firmicutes)	*Geoth_0008/Geoth_0007*	*Proteobacteria*, *Firmicutes*
		A*6.T13	Phage_integrase.Zeta_toxin		acidovorax_sp_12322_1 (Proteobacteria)	*AS359_01855/AS359_01860*	
					arcobacter_butzleri_l348 (Firmicutes)	*AA20_09385/AA20_09380*	

Many x antitoxins are annotated as nearest to Pfam HTH_3 (A*1 and A*8) and RHH_1 (A*27) features, for instance in the following pairing types: HigB_toxin.HTH3, HipA_C.HTH_3, HTH_3.HipA_C, ParE_toxin.HTH_3, RelE.HTH_3 and RHH_1.ParE_toxin. These HTH_3 and RHH_1 Pfam annotations are too general to directly infer functional clues for these putative new antitoxin families but they are good candidates to discover new antitoxin families. Each of the different popTx groups derived from these HTH_3 and RHH_1 combinations would require further characterization based on cognates alignments and structural analyses for example. Some other interesting x antitoxins are the ones with nearest Pfam annotations of Colicin_Pyocin (A*190, as in Colicin_Pyocin.YafQ_toxin—.A*190.T4), VraX (A*371, as in VraX.PemK_toxin—A*371_T143, specific to *Staphylococcus*), Glyoxalase (A*77, as in YafQ_toxin.Glyoxalase—T32.A*77), Antirestrict (A*237, as in Antirestrict.CbtA_toxin—A*237.T3) and Response_reg (T5, as in Cpta_toxin.Response_reg—T5.A*2). VraX (IPR035374) and Glyoxalase (IPR004360) are both involved in antibiotics resistance pathways. The VraX-like putative antitoxins seem to originally be derived from a phage protein. Intriguingly, the VraX.PemK pair is not found in the reference *Staphylococcus aureus subsp*. *aureus NCTC 8325* while it is present in other *S*.*aureus* strains (S4 Fig). Colicin_Pyocin and Response_reg families could potentially give some clues in the evolution of the TAS. The Colicin_Pyocin (IPR000290) family contains the immunity proteins and/namely members of the effector-immunity system, which is a two-component genetic system (TCS) similar to the TAS but where both cognates are secreted in order to protect the bacteria itself and its clonemates [50]. Response_reg (IPR001789) belongs to another two-component genetic system called “two-component signal transduction system”, which also presents similarities with the TAS. Previous publications have already suggested potential interplay and/or homology between different TCS [51,52]. Finally, annotations from other x antitoxins indicate that many more popTx could be promising candidates: Ap_endonuc_2 (as in AP_endonuc_2.ParE_toxin) and Phage_integrase (as in CcdB.Phage_integrase, Phage_integrase.PemK_toxin or Phage_integrase.Zeta_toxin). These two candidates highlight the link between the TAS and the phages. More investigation will be needed to confirm these candidates as functional new antitoxin families.

## Discussion

We believe that the strength of TASmania is its discovery-oriented feature. Although this may lead to unwanted false positives, it also allows for the identification of candidate TAS in species previously described as not containing any TAS loci. Typically, the *Prochlorococcus marinus* and *Mycoplasma* are good examples to show the advantage of TASmania. Indeed, while no hit is predicted using TAfinder, TASmania shows that various *Mycoplasma* assemblies harbour putative type II (*e*.*g*., the TA pair *D500_0109/D500_0110* in *Mycoplasma feriruminatoris*, which corresponds to a Pfam YafQ/RelB-like pair) and type IV (*e*.*g*., *MAGb_3900/MAGb_391*0, an AbiEii/AbiEi_4-like pair in *Mycoplasma agalactiae 14628*) hits. In addition, TASmania identifies several putative TAS (including many orphan loci) in various *Prochlorococcus marinus* assemblies, which would need further investigation before validation as type II, and also some less clear TAS types like *P9303_20011/P9303_20021* pair in *Prochlorococcus marinus str mit 9303* (similar genes also in other related assemblies) that correspond to a PIN/Clp-like pair. Intriguingly the next neighbour gene *P9303_20031* is also a Clp protease. Overall, TASmania data indicate that even species previously considered as “TAS-free” in the literature might actually contain TAS loci, but whether these are expressed *in vivo* and are biologically functional would require to be investigated in further experimental analysis.

By avoiding any assumption in the TA protein length and the type of operon—TASmania includes orphan TA loci and TAS hits from multigene pseudo-operons—our database opens up to new TAS families and possible networks. In parallel, we use our large database to apply a meaningful analysis of the biology of the TAS by looking at their organisation in pairs. Our results highlight the modularity of the TA cognates and the issues raised by the conventional misleading family annotations of the TAS. Currently TASmania has three main limitations: i) due to our discovery approach, we suspect that the false positive rate might be high, but it is difficult to assess ii) the downside of automated clustering methods in general iii) the absence of the phage genomes (but prophages and plasmids are included). One should also note that TASmania can contain putative type V and type VI as “side hits”, although these were not mined for purposely. These hits correspond to T or A mined from type I-IV HMM profiles, but due to the modularity, plasticity and the rapid evolvability of the TAS [4,5,38], they can be found in type V-VI. Beside the discovery of uncharacterized TAS missed by alternative sources, TASmania can provide valuable help in the experimental design step. Indeed, the frequent presence of multiple TAS within same genomes, including orphan loci, raise the issue of potential (positive and/or negative) interference in *trans*. By providing an *in silico* updated map of putative TAS, TASmania offers the possibility to consider a maximum of potential interferences of TAS in *trans* when designing an experiment, and to compare this with other strains of interest. Ideally, RNA-seq data should be combined with the TAS *in silico* annotation in order to get an accurate landscape of TAS. TASmania is very powerful thanks to its large number of assemblies (>41K), which has never been proposed so far. Some of TASmania’s potential applications are phylogenetics and phenotypic comparisons of different isolates. For instance, TASmania can help in making comparative studies by more accurately mapping putative TA loci in *E*.*coli* strains with various pathogenicity [53], or in *Endozoicomonas* sequenced strains from different ecosystems [54], highlighting how this could link to the associated hosts (our own unpublished data).

### Conclusion

TASmania is a new resource for the discovery of toxin-antitoxin in known bacterial genomes. Even though it is based on existing protein domain descriptions, its flexibility allows for the uncovering of potential new combination of pairs and totally new families of toxins and/or antitoxins using a guilt-by-association strategy. The experimental validation *in vivo* of several predicted TAS confirms the potential of this resource for the identification of TAS.

## Methods

### Building reference TAS HMM profiles

The global strategy is to build an updated list of toxin and antitoxin HMM profiles and scan a local version of the EnsemblBacteria database (N>41K assemblies) with thoses HMM profiles. To achieve this, we have downloaded EnsemblBacteria (release 33, November 2016) [55], updated its InterPro (IPR) (version Nov 2016) [56] annotation and applied a pseudo-operon annotation with arbitrary definition where a maximal intergenic distance of 100 bp is applied, as shown in Fig 1.

**A) Building the reference TAS IPR list.** An initial reference TAS IPR database is built as following. Based on a keyword search in UniProtKb ("toxin+antitoxin"), a set of N∼44K proteins is extracted. These hits are filtered to keep only the hits corresponding to the Bacteria superkingdom (id = 2) and annotated with at least 1 IPR, giving N∼37K proteins. We extract the IPR annotations of these proteins and we obtain N = 733 unique IPR derived from this set. In order to help in the selection of the TAS-specific IPR, we fetch the IPR detailed descriptions from EBI to manually review whether each one of these 733 would be included to the initial reference TAS IPR database. We obtain a total of N = 80 reference TAS IPR list, of which N = 45 correspond to toxin and N = 35 to antitoxin IPRs.
**B) Updating the IPR annotation of EnsemblBacteria initial database**. Meanwhile, we update the IPR annotation of our database. Indeed, although the gene annotation for domain features (Pfam, PROSITE etc) is accurate, we discovered that their equivalent IPR was partially missing in our database (EnsemblBacteria core 33 release 33). Based on the InterPro release 61 that maps each IPR identifier to corresponding domains features (Pfam etc), we update the IPR annotations of all the genomes by linking their domain features with the new IPR release.
**C) Identify an initial set of proteins with an IPR mapping to reference TAS IPR**. The reference TAS IPR list is used to identify an initial set of proteins in the database and gives N = 120’416 putative toxins and N = 90’048 putative antitoxins (both unique proteins sequences).
**D) Building the HMM profiles.** The TAS IPR annotated toxins (same protocol for antitoxins) proteins are then i) clustered with MMSeqs2 [57]; ii) a MSA is calculated for each cluster size greater than 10 unique protein sequences (ClustalO version 1.2.4) [58]; iii) the MSA of each cluster is used to build an HMM profile (HMMER3 version 3.1b2 February 2015 http://HMMer.org).
**E) HMM search.** Finally these HMM profiles are searched against the whole N = 41’604 proteomes, as follows: hmmsearch <hmm_profiles_database> <proteome.fasta> from HMMER3 (default settings).

### Combining HMM profiles into clusters

In parallel, we perform HMM profiles comparison in order to reduce the number of profiles, using the Profile Comparer program PRC (v1.5.6) [59]. Combining the PRC results with the NetworkAnalyzer [60] in CytoScape (3.5) [61] network analysis, we select the first PRC E-value of 10^−12^ where the number of connected components (CC) (*i*.*e* clusters of HMM profiles) is reaching the plateau. For clarity and continuity with previous TAS annotations found in the literature, each TASMANIA cluster identifier is given the nearest corresponding Pfam family names (release 31.0) to which the TAS scientific community is used to. The “nearest” Pfam annotation is performed as follows: using the PRC program for profile-profile comparison (default settings), each TASmania HMM profile is scanned against Pfam database. The best Pfam profile match for each TASmania HMM profile (i.e., the lowest E-value) is selected and the identifier of this Pfam annotation is used as the Pfam equivalent of the given TASmania HMM profile. On top of the HMM profile annotation, the TASmania clusters are also attributed a Pfam annotation. For each TASmania cluster we attribute the common profile Pfam annotation when there is no ambiguity. In cases of heterogeneity (more than one Pfam annotation per cluster), the Pfam match with the smallest E-value is selected. But in all cases, the individual Pfam annotation of each TASmania HMM profile is kept and shown in S2 Table for methodology coherence. We used the word”nearest” to emphasize the potential issues of such equivalences.

The final TASmania database contains: i) the putative hits from the HMM scan; ii) the genes annotated with a reference TAS IPR and that were filtered out due to the small size of their proteins clusters (less than 10 unique sequences) when building the HMM profiles; iii) the guilt-by-association “x” cognates (see S5 Fig). We also add an extra annotation of the putative TAS hits by analysing the cis-occurrence—within a same pseudo-operon—of toxins and antitoxins clusters: we call these T<->A clusters associations “popTA” groups. To construct these popTAs we first define the pseudo-operon structures using a relaxed model containing one, two or more genes. Our pseudo-operon model is simply based on an arbitrary intergenic distance -100nt < = D < = +100nt between adjacent genes oriented in the same direction (strand), keeping in mind that there is no "one-size-fits-all" D value. We selected the arbitrary value of 100nt based on some previous studies of intergenic distances distributions [62]. The pipeline is summarized in the Fig 1. The popTA sequences comparisons in Fig 9 are done with ClustalO, the MSA plots with Jalview (2.9.0b2) [63] and the HMM profiles of the MSA are plotted with Skylign [64].

### Experimental validation of putative TAS hits

Plasmid constructs. Plasmid pLAM12 [65] has been described elsewhere. The eleven putative new toxins identified by TASmania were PCR amplified using primers from S7 Table and cloned in pLAM12 under the control of an acetamide inducible promoter. Cloning was performed using appropriate restriction enzymes or by In-Fusion methodology (Clontech), as indicated in S7 Table. Constructs were sequence verified using primers pLAM-For 5’- ACCCTCCACCGGCCGCGCTC and pLAM-Rev 5’- TGGCAGTCGATCGTACGCTA. For toxins that affected *M*.*smegmatis* growth, their respective toxin-antitoxin operons (six in total) were then PCR amplified and cloned into pLAM12, using appropriate primers from S7 Table.

*In vivo* growth assay. The pLAM12-based constructs were first electroporated in *Strain M*.*smegmatis* MC^2^155 (strain ATCC 700084). Following 3 h incubation at 37°C in LB medium + tween 80 (0,05%), 1/100 of the transformants were directly plated on LB agar supplemented with kanamycin (20 μg/ml) and acetamide (0,2%). Plates were incubated 3 days at 37°C.

Note that *Rv0229c* only showed a tiny but reproducible effect on *M*.*smegmatis* growth when expressed alone (Fig 5). Therefore, we decided to test it within the context of its operon as well. The effect of the putative antitoxin *Rv0230c* was hardly detectable (S6 Fig), indicating that *Rv0229c/Rv0230c* may not be a functional TA system when expressed in *M*.*smegmatis*.

### New antitoxin families discovery: Guilt-by-association method

Similar to the popTAs analysis performed on the canonical TA/AT hits previously, we pool all the “x” protein sequences, cluster them with MMseqs2, make an MSA of each cluster, build an HMM profile for each protein cluster, and compare and cluster the HMM profiles (N = 805) with PRC and Cytoscape. We dub these putative antitoxin HMM clusters as TASMANIA.A*n (A*n) (N = 536 at E-value = 10^−5^). After Pfam annotation of these putative antitoxin clusters, we perform a semi-automated curation to discover new antitoxin families. One criterion of selection we applied is that the nearest Pfam annotation of the “x” antitoxin should not belong to known antitoxin families (*e*.*g*., ParD_antitox, CcdA, CbeA_antitoxin, MazE_antitoxin, PhdYeFM_antitox, CopG_antitoxin, AbiEi, VAPB_antitox). We then go further in stringency by selecting only pairs whose T toxin cognate had an HMM E-value below 10^−20^ and we thus obtain N = 222 xT/Tx combinations. We find that 27 popTx contain putative new antitoxin protein families worth investigating, since they are conserved up to high stringency.

## Supporting information

S1 Fig Bias in EnsemblBacteria phylum content. The *Proteobacteria* and *Firmicutes* are overrepresented in the database. The weight of each phyla will be taken into account when counting the hits in the popTA analysis in particular.
(TIF)

S2 Fig Phylum distribution of the species that are the most enriched in TA: *Cyanobacteria* are the winners. Top 20 (**A**), 50 (**B**), 100 (**C**) and 200 (**D**) species enriched in TA correspond mainly to *Cyanobacteria*. The hits counts of each phylum has been corrected according to the weight of the different phyla in the database. Only the canonical AT/TA hits, with an HMM E.value below 1E-04, from two-genes pseudo-operons have been taken into account.
(TIF)

S3 Fig Complete co-occurrence heatmaps of toxin and antitoxin clusters within two-genes pseudo-operons (popTAs). (A) Antitoxin clusters in A->T orientation, and their relation to toxin clusters. (B) Antitoxin clusters in T->A orientation, and their relation to toxin clusters. (C) Toxin clusters in A->T orientation, and their relation to antitoxin clusters. (D) Toxin clusters in T->A orientation, and their relation to antitoxin clusters. Each heatmap should be read from column to rows, in order to evaluate the modularity of a given cluster ID.
(TIF)

S4 Fig MAUVE alignment of some *Staphylococcus aureus* strains at a putative new antitoxin family VraX, as in VraX.PemK_toxin popTx. Interestingly, the TASMANIA.A*371_TASMANIA.T143 popTx is missing in *S*.*aureus subsp*. *aureus NCTC 8325* (top yellow box), where, although the VraX equivalent locus seems to be present (SAOUHSC_02236), its neighbour gene (SAOUHSC_02237, a phage protein) is not given as toxin cognate by TASmania.
(TIF)

S5 Fig Snapshot of TASmania web server’s content. (TIF)

S6 Fig Rescue test of Rv0230c/Rv0229c. (TIF)

S1 Table Reference TAS InterPro IPR list. This arbitrary starting list is used to build the IPR annotated proteins databases that are later clustered and aligned when building the HMM profiles.
(XLSX)

S2 Table Toxins and antitoxins HMM profiles description. Nearest Pfam annotation of TASmania HMM profiles and clusters.
(XLSX)

S3 Table List of the TASmania hits missed by TAfinder. In the four reference genomes.
(XLSX)

S4 Table List of the TAfinder hits missed by TASmania. In the four reference genomes. In red are highlighted the 2 genes that seem to be false negative missed by TASmania.
(XLSX)

S5 Table List of observed partners of toxins and antitoxins clusters in popTAs. Only canonical AT/TA two-genes pseudo-operons are being considered.
(XLSX)

S6 Table Clusters modularity and putative popTA crosstalks. Examples of A74 and A12 clusters in different genomes. Only canonical AT/TA two-genes pseudo-operons are being considered.
(XLSX)

S7 Table *M*.*tuberculosis* toxins and TA operons cloned into pLAM12 vector. (XLSX)
