# Supplementary figures and images for: TASmania: A bacterial Toxin-Antitoxin Systems database
Source: PLoS Comput Biol. 2019 Apr 25;15(4):e1006946. doi: 10.1371/journal.pcbi.1006946 (PMC6504116; doi:10.1371/journal.pcbi.1006946)

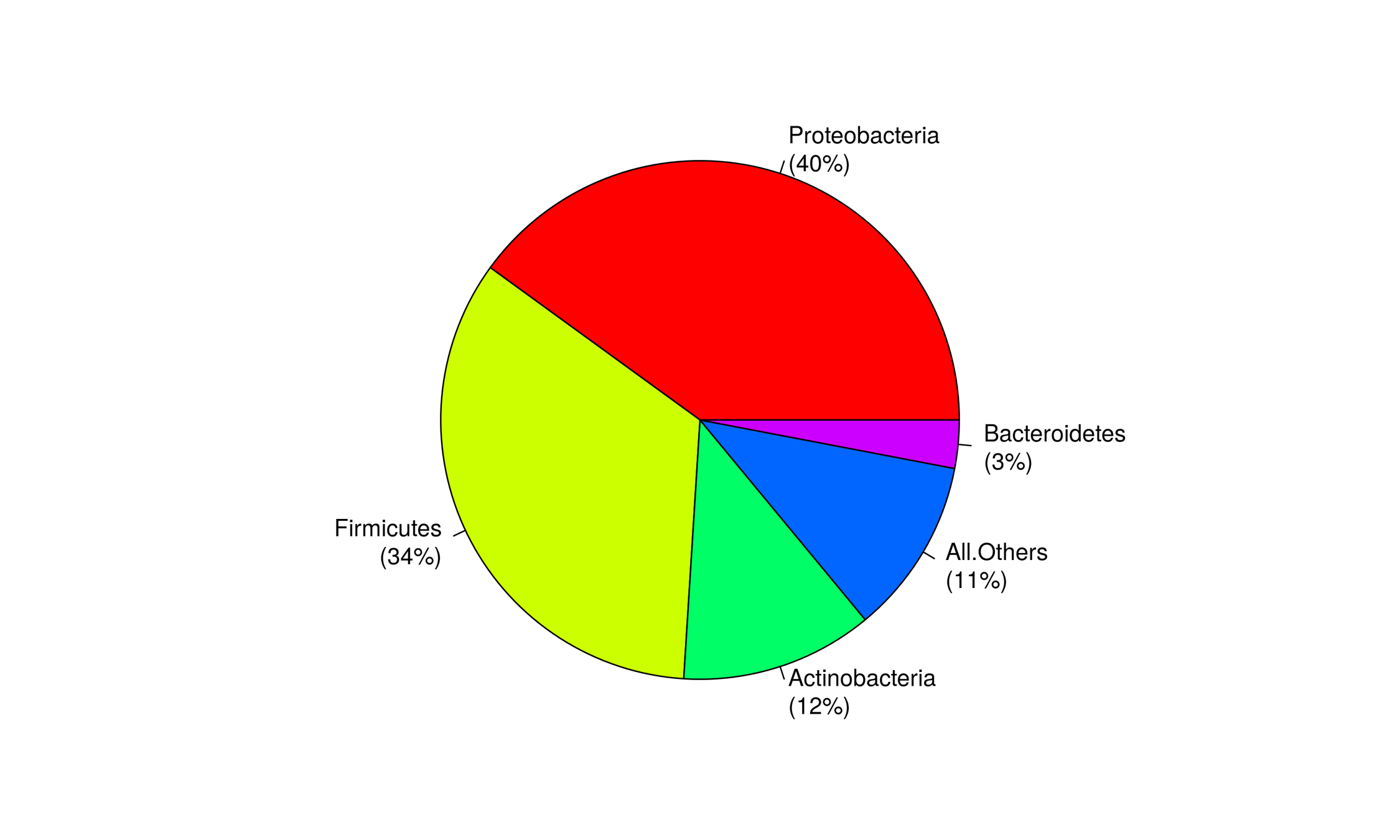

Supplement: S1 Fig — The Proteobacteria and Firmicutes are overrepresented in the database. The weight of each phyla will be taken into account when counting the hits in the popTA analysis in particular. (TIF) [file pcbi.1006946.s001.tif]

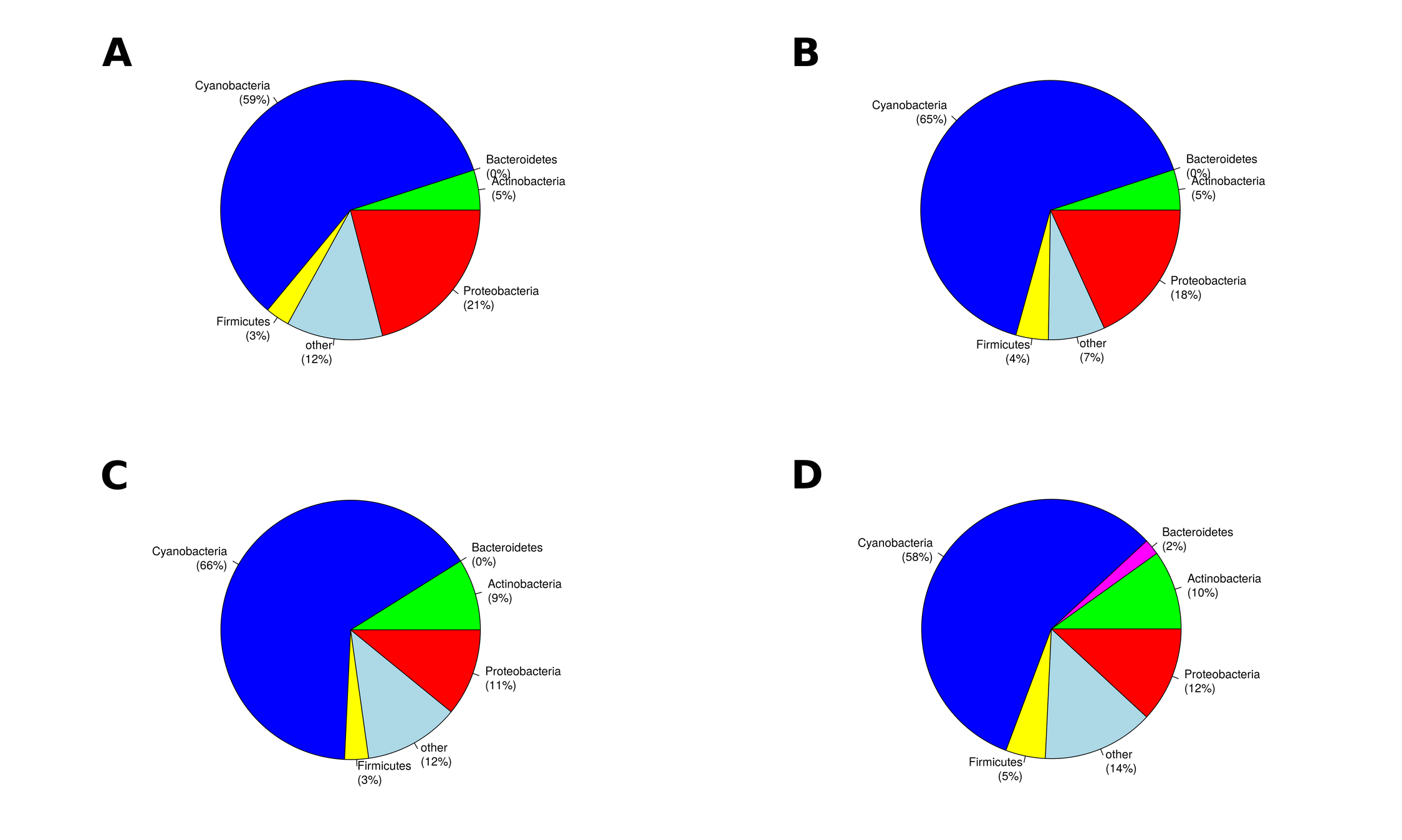

Supplement: S2 Fig — Top 20 (A), 50 (B), 100 (C) and 200 (D) species enriched in TA correspond mainly to Cyanobacteria. The hits counts of each phylum has been corrected according to the weight of the different phyla in the database. Only the canonical AT/TA hits, with an HMM E.value below 1E-04, from two-genes pseudo-operons have been taken into account. (TIF) [file pcbi.1006946.s002.tif]

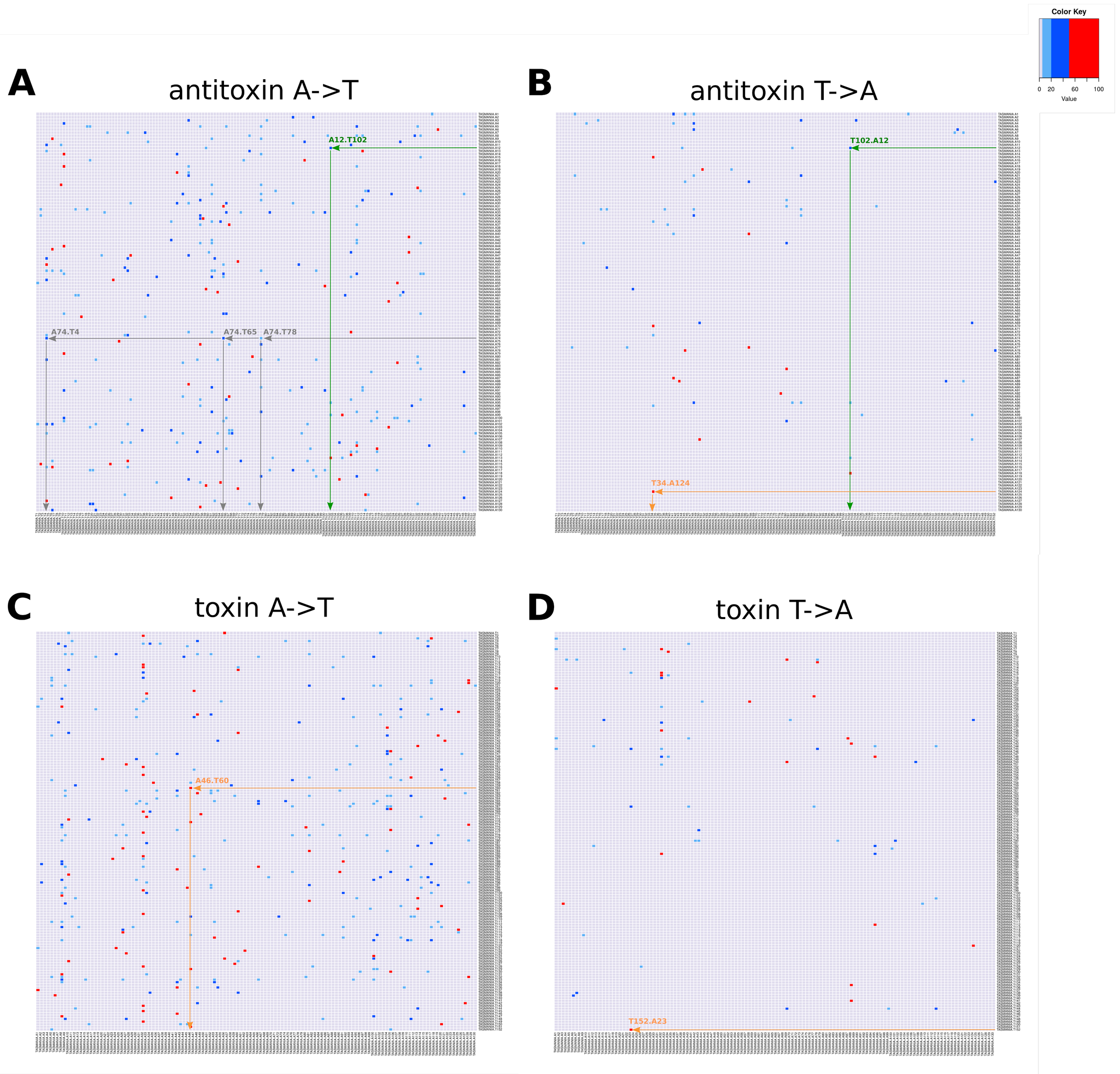

Supplement: S3 Fig — (A) Antitoxin clusters in A->T orientation, and their relation to toxin clusters. (B) Antitoxin clusters in T->A orientation, and their relation to toxin clusters. (C) Toxin clusters in A->T orientation, and their relation to antitoxin clusters. (D) Toxin clusters in T->A orientation, and their relation to antitoxin clusters. Each heatmap should be read from column to rows, in order to evaluate the modularity of a given cluster ID. (TIF) [file pcbi.1006946.s003.tif]

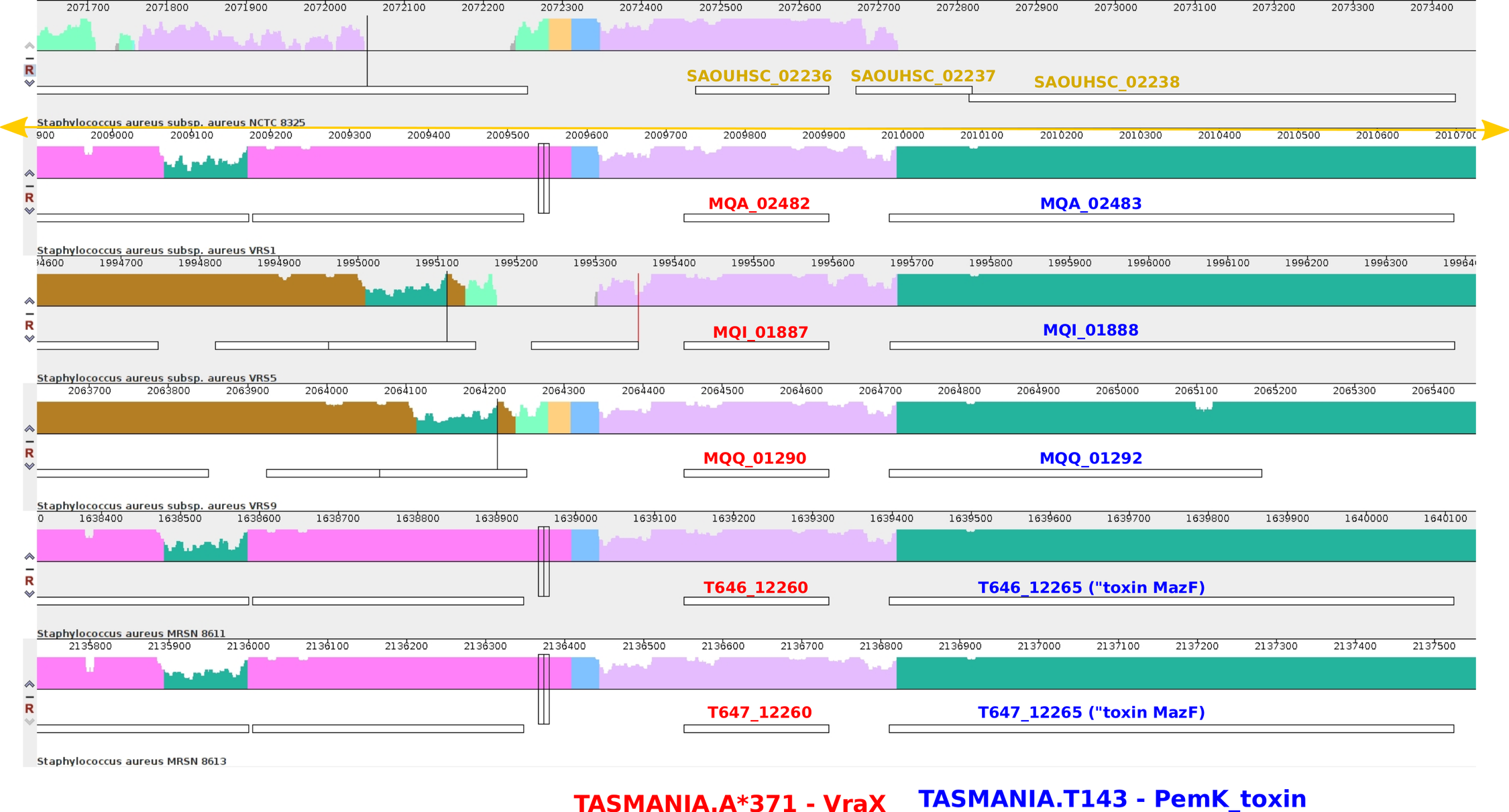

Supplement: S4 Fig — Interestingly, the TASMANIA.A*371_TASMANIA.T143 popTx is missing in S.aureus subsp. aureus NCTC 8325 (top yellow box), where, although the VraX equivalent locus seems to be present (SAOUHSC_02236), its neighbour gene (SAOUHSC_02237, a phage protein) is not given as toxin cognate by TASmania. (TIF) [file pcbi.1006946.s004.tif]

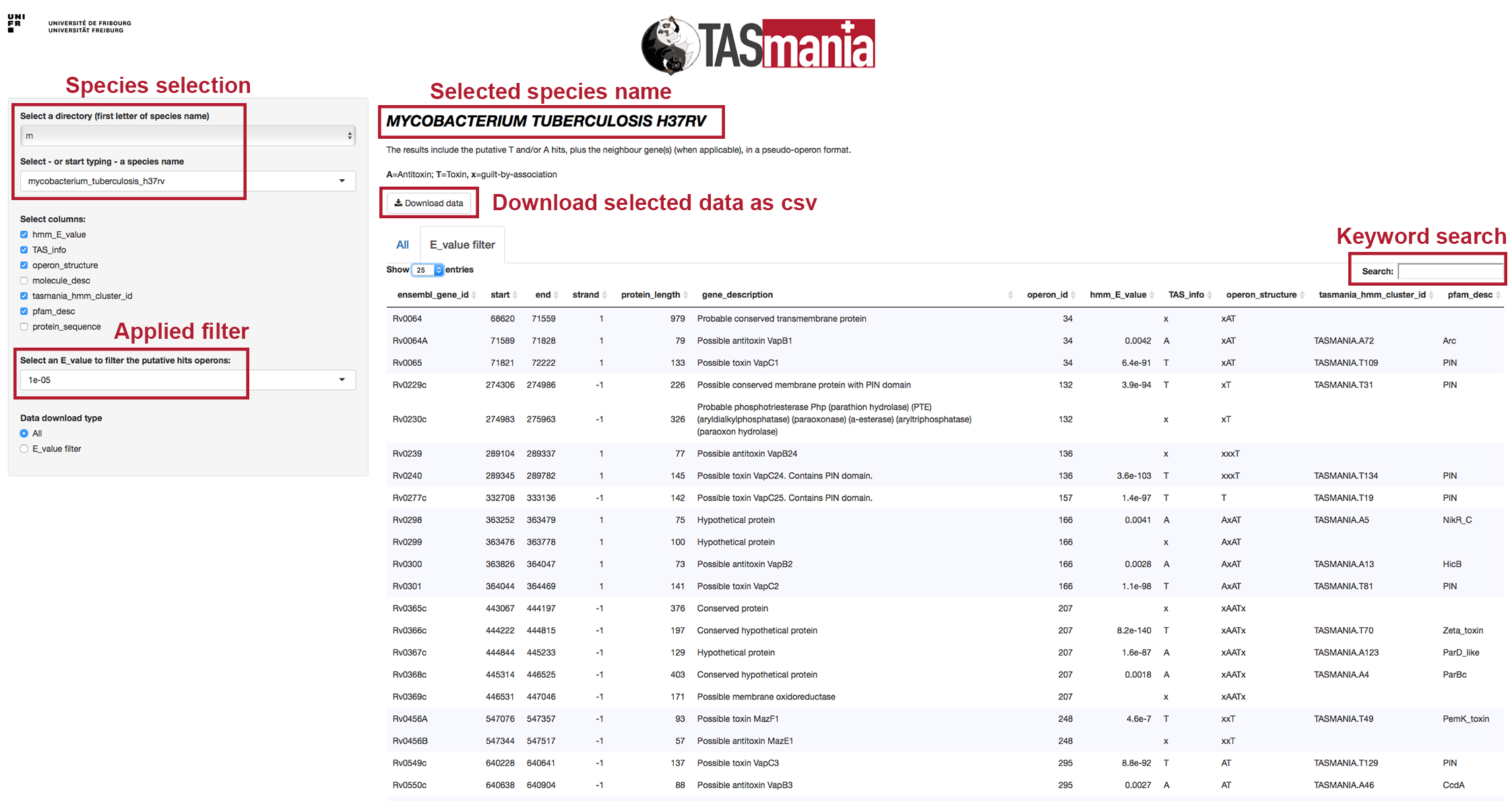

Supplement: S5 Fig — (TIF) [file pcbi.1006946.s005.tif]

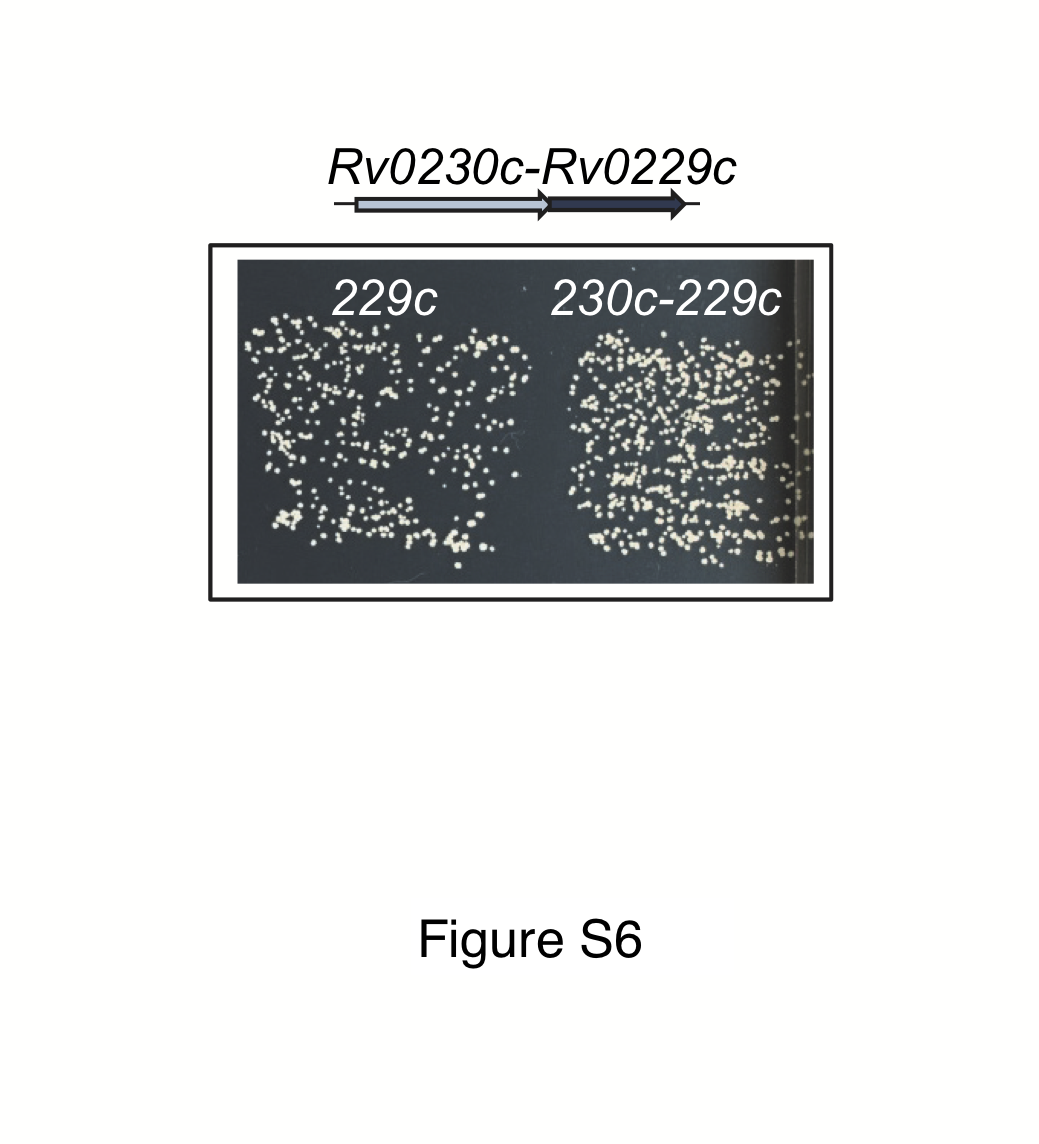

Supplement: S6 Fig — (TIF) [file pcbi.1006946.s006.tif]
